# No evidence from a negative mood induction fMRI task for frontal functional asymmetry as a suitable neurofeedback target

**DOI:** 10.1038/s41598-023-44694-3

**Published:** 2023-10-16

**Authors:** Jingying Zhang, Vera Eva Zamoscik, Peter Kirsch, Martin Fungisai Gerchen

**Affiliations:** 1 Department of Clinical Psychology, Central Institute of Mental Health, University of Heidelberg/Medical Faculty Mannheim, Mannheim, Germany; 2 Department of Psychology, University of Heidelberg, Heidelberg, Germany; 3 Bernstein Center for Computational Neuroscience Heidelberg/Mannheim, Mannheim, Germany

**Keywords:** Psychology, Medical research, Magnetic resonance imaging

## Abstract

Frontal functional asymmetry (FA) has been proposed as a potential target for neurofeedback (NFB) training for mental disorders but most FA NFB studies used electroencephalography while the investigations of FA NFB in functional magnetic resonance imaging (fMRI) are rather limited. In this study, we aimed at identifying functional asymmetry effects in fMRI and exploring its potential as a target for fMRI NFB studies by re-analyzing an existing data set containing a resting state measurement and a sad mood induction task of n = 30 participants with remitted major depressive disorder and n = 30 matched healthy controls. We applied low-frequency fluctuations (ALFF), fractional ALFF, and regional homogeneity and estimated functional asymmetry in both a voxel-wise and regional manner. We assessed functional asymmetry during rest and negative mood induction as well as functional asymmetry changes between the phases, and associated the induced mood change with the change in functional asymmetry. Analyses were conducted within as well as between groups. Despite extensive analyses, we identified only very limited effects. While some tests showed nominal significance, our results did not contain any clear identifiable patterns of effects that would be expected if a true underlying effect would be present. In conclusion, we do not find evidence for FA effects related to negative mood in fMRI, which questions the usefulness of FA measures for real-time fMRI neurofeedback as a treatment approach for affective disorders.

## Introduction

Frontal functional asymmetry (FA) has been proposed as a potential target for neurofeedback (NFB) training for mental disorders but most FA NFB studies used electroencephalography (EEG) while the investigations of FA NFB in functional magnetic resonance imaging (fMRI) are rather limited. In this study, we aimed at identifying functional asymmetry effects in fMRI and exploring its potential as a target for fMRI NFB studies to address pathological processes and symptoms that are associated with this process^1^. Over the last decade real-time fMRI (rtfMRI) NFB has emerged as a sophisticated methodology to target spatially precise cortical signals^2–5^, and its clinical application is now explored extensively^6,7^. One of the main challenges for the development of rtfMRI NFB approaches is the identification of suitable target brain processes, and FA is one of the potential target processes that have been proposed to have utility in NFB training based on its association with emotional and motivational processes^8^.

Frontal asymmetry between the two hemispheres of the brain is a common concept for understanding the function of the human brain, which is also almost ubiquitous in lay theories. While the two hemispheres of the brain are similar in size and weight, an asymmetry is evident in both anatomical (i.e. size, surface area, and cortical thickness) and functional domains^9^. Frontal functional asymmetry (FA) can be regarded as both a trait measure, which is relatively stable across time, as well as a state-dependent response to environmental stimuli. The trait FA is mostly examined at rest and reflects intra-individual differences. It is related to other psychological traits (such as depression, anxiety, anger, and behavioral activation^10^) and can modulate state response to emotional stimulus^11–14^. The state FA is usually measured with emotionally evocative tasks (for example emotionally evocative film clips, anger elicitation tasks, insult stimuli tasks, reward or punishment incentive tasks^13^), and some proposed that the FA may be an interaction between the emotional salience and the emotion predisposition thus emotional tasks may reveal FA better than the rest state^14–16^. Both concepts are relevant in NFB training: When we want to reduce a trait that might be a vulnerability factor for mental disorders, we should target trait FA. If we want to train people to modulate their emotional state, we should target state FA.

Frontal asymmetry has been intensively studied in its relationship with emotion, motivation, personality, and psychopathological constructs (e.g.^17–19^). According to the approach-withdrawal motivational/emotional model^12,20–22^, left-sided frontal activity is assumed to be related to the approach system, which elicits positive emotion, approach-related motivation, and goal-directed behavior; while right-sided frontal activity is assumed to be associated with the withdrawal system, which elicits negative affect, withdrawal-related motivation and behavior^23^. Based on this model, the greater right side frontal activity has been proposed as a risk factor for the development of affective disorders^24–27^, and has been used as a target in NFB. For example in depression, several studies have suggested that modulating functional asymmetry in NFB can reduce depressive symptoms (e.g.^28–30^). Notably, most of these NFB studies used the EEG technique^31,32^.

With MRI, studies have investigated frontal structural asymmetry as a trait ^9,33–35^, and explored its association with mental disorders^36^. While investigations of FA with fMRI and the application of FA rtfMRI NFB are rather limited. Existing fMRI FA studies have supported the approach-withdrawal model (e.g.^37–39^), and some researches have explored fMRI FA in affective disorders, for example, Herrington et al.^40^ found that both depressed patients and healthy participants showed leftward lateralization for pleasant words, while depressed patients showed more right-lateralized activation for unpleasant words; Li et al.^41^ compared ReHo of 16 depressive patients with 15 healthy subjects, and the results showed the differential ReHo values were mainly located in the right hemisphere.

For further progress in the development of fMRI NFB approaches it is pivotal to characterize and validate possible processes that could be used as targets. In this regard, it is especially interesting to identify cross-modal NFB target processes that could be addressed with different technologies like EEG and fMRI and would allow comparisons between the modalities.

Some attempts have been made to combine the EEG and fMRI techniques in FA NFB studies. In a series of studies, Zotev et al.^42–45^ conducted combined real-time fMRI-EEG NFB and found associations between the blood-oxygen-level-dependent (BOLD) signal in the amygdala and frontal EEG power asymmetry in healthy participants^42^ and in patients with major depressive disorder (MDD)^43,44^. Zotev et al.^45^ also conducted another frontal alpha asymmetry EEG NFB study with simultaneous fMRI in MDD and reported temporal correlations between the frontal alpha asymmetry and BOLD activation for brain regions involved in emotion regulation, but not with BOLD asymmetry. A few further studies examined the convergence of EEG asymmetry and fMRI brain activation. Gorka et al.^46^ investigated their convergence during reward anticipation and found that increased relative left frontal EEG activity is associated with increased left anterior cingulate cortex (ACC)/medial prefrontal cortex (mPFC) and left orbitofrontal cortex (OFC) activation. Morys et al.^47^ used fractional amplitude of low-frequency (fALFF) to measure functional asymmetry in resting-state fMRI, but found no correspondence between EEG asymmetry and whole-brain fALFF asymmetry in the approach/avoidance context, and they assumed this incongruence may due to huge differences in the physiological basis of the signals.

To further investigate this topic and extend the fMRI literature on functional asymmetry, we examined fMRI functional asymmetry effects by re-analyzing an existing fMRI data set from Zamoscik et al.^48^ that was obtained to investigate the neural correlates of negative mood induction in remitted major depressive disorder (rMDD) and to assess their predictive value for the future course of depressive symptoms. In this study, two groups of rMDD patients and healthy controls (HC) matched for age, gender, and education underwent a resting state scan and a sad mood induction task in which keywords about personal negative life events were shown and sad music was played. The task successfully induced sad mood in both groups (and to a greater extent in the rMDD group) as shown in the previous study^49^. With this data set, an association between respiration pattern variability, mood, and depression on the behavioral and neural levels was found (see further details in^48^). This data set is ideally suited for investigating changes in functional asymmetry associated with negative mood. Although it was not acquired for this purpose, if we would have designed a study for addressing our research question, we would likely have come up with a very similar study design.

Technically, the study applied a low frequency experimental design which allows to analyze the data of both the rest and the mood induction phase with methods that are usually applied to resting state data. Therefore, we investigated functional asymmetry based on three analysis methods that are suited for the data: amplitude of low-frequency fluctuations (ALFF)^50^, fractional ALFF (fALFF)^51^, and regional homogeneity (ReHo)^52^. Functional asymmetry values based on ALFF and fALFF represent the asymmetry in the amplitude of low-frequency changes in the BOLD signal, while ReHo functional asymmetry reflects the asymmetry of the local synchrony of the BOLD signal.

This approach enables us to assess the trait functional asymmetry at the resting phase, the state functional asymmetry at the sad mood induction phase, and the moderating effect of traits on states (by comparing the functional asymmetry change over phases of the two groups), and associate the induced change in mood with the change in functional asymmetry. All of these effects can be investigated within the rMDD and HC groups as well as compared between the groups. If there is an association between fMRI functional asymmetry and negative emotions, we should be able to detect systematic signatures of these effects with our procedures and suggest its potential as a target for fMRI NFB studies.

## Methods

### Sample

For this paper, we re-analyzed already existing and preprocessed data from Zamoscik et al.^48^. We re-analyzed the main sample of 30 rMDD and 30 matched HC. The originally acquired sample comprised 64 individuals of which 4 individuals were excluded due to incidental anatomical findings.

Participants with rMDD were enrolled if they had at least two previous major depressive episodes or a previous chronic major depressive episode of at least two years duration according to DSM-IV and were in partial or full remission for at least the previous two months. Participants were excluded if they fulfilled the criteria of bipolar and psychotic disorders, substance dependence, current substance abuse, current obsessive–compulsive, posttraumatic stress, and eating disorders, have contraindications for the MRI (including hypertension, heart diseases and surgeries, and other severe illnesses). Healthy participants fulfilled the same criteria but did not have a history of depression and were enrolled if they matched the patients by age, gender, and education level.

The study was approved by the local ethics committee of the Medical Faculty Mannheim of Heidelberg University. All methods were performed in accordance with the relevant guidelines and regulations.Written informed consent was obtained from all participants.

### Data acquisition

Participants went through a six-phase experiment, each phase lasts 4.5 min: two resting states, two sad mood inductions, one rumination phase and one distraction phase (the order of the rumination and distraction phases were counterbalanced). For the present paper, only the first resting state and the first mood induction phases were analyzed. The Positive and Negative Affect Scale (PANAS)^53^ was measured before and after each phase with a built-in keypad to assess the positive and negative affect. In the present study, we used the initial resting state and the first sad mood induction phase, and the PANAS score after these phases.

fMRI data were acquired with a 3 T Trio TIM Scanner with a 12-channel head coil (Siemens Medical Systems, Erlangen, Germany). T1-weighted structural images were obtained with repetition time (TR) = 2.3 s, flip angle (α) = 9°, echo time (TE) = 3.03 ms, 192 slices, slice thickness = 1 mm, voxel size = 1 × 1 × 1 mm^3^, FOV = 256 mm^2^. In each phase 180 T2* weighted echo planar imaging (EPI) sequences were acquired with TR = 1.5 s, α = 80°, TE = 28 ms, 24 slices, slice thickness = 4 mm, voxel size = 3 × 3 × 4 mm^3^, FOV = 192 mm^2^. Respiration and heart rate were recorded at 50 Hz with the scanner built-in equipment.

### Negative mood induction task

Before the fMRI session, participants were shortly interviewed about three negative life events and provided keywords that reminded them of these events (for example ‘breakup Nina’, ‘death granny’, ‘cellar asylum’, ‘New Year’s Eve 2010’). In the scanner, the task was conducted using Presentation software package (version 18.1; www. neurobs.com). During the resting state, participants were asked to keep their eyes open, the keywords ‘Rest 1’, ‘Rest 2’, and ‘Rest 3’ were shown for 4.5 min (1.5 min for each), and background pink noise was presented. During the sad mood induction phase, the individual pre-recorded keywords for personal negative life events were presented (each for 1.5 min, thus 4.5 min in total) and sad background music (parts of Adagio in g-minor by Albinoni) was played. Participants were instructed to focus on the self-provided keywords and think about themselves in the situation.

### Data analysis

#### Behavior

Demographic characters (age and gender) were analyzed with IBM SPSS22 (SPSS Inc., Chicago, Illinois, USA). Independent t-tests and chi-square tests were conducted to compare the distribution of the categorical variable (gender) and continuous variable (age). PANAS scores were analyzed with Matlab (R2021b; MathWorks Inc., Sherborn, Massachusetts, United States). Hedge's g and 95% confidence interval (CI) were estimated based on the t-value to assess the effect size of group differences and time differences^54^. The correlation between PANAS scores and functional asymmetry values was calculated by second level SPM regression analyses. The statistical significance threshold is *p* < 0.05.

#### Imaging analyses

fMRI image preprocessing and analyses were conducted with SPM8 v5236 (Wellcome Trust Centre for Neuroimaging, University College London, UK).

#### Preprocessing

The first 20 images of each phase were removed from analyses, resulting in 160 volumes in each task and resting state data set that was analyzed. Data were corrected for physiological artifacts using a Matlab software (Aztec, https://www.neuromri.nl/2015/12/14/aztec-cardiorespiratory-correction-software-for-functional-mri/)^55^ including a high-pass filter of 1/512 Hz. The images were motion corrected, slice time corrected (with the 13th slice as reference), normalized to an EPI template, and smoothed with a Gaussian kernel of 9 mm. Wavelet despiking^56^ was applied for motion-correction. It identifies and removes non-stationary events caused by head movement in the fMRI time series without the need for data scrubbing. This approach is not limited by the temporal resolution of movement parameter information and is able to characterize non-stationary event coefficients in multiple frequency bands. This capability allows us to motion-correct low-frequency data and remove artifacts only in the frequencies in which they occur, but leaving the other frequencies intact.

#### ALFF and fALFF

ALFF is computed by the average square root of a power spectrum across 0.01–0.08 Hz at a voxel, it represents the deviation of the spontaneous low-frequency fluctuations (LFF) of BOLD signal^50^. It is a reliable measure to index spontaneous brain activity in the resting state, and due to its character of unconstrained by experimental design and task-related performance confounds, it can identify the effects of mental conditions on spontaneous brain activity. fALFF was introduced by Zuo et al.^51^ to reduce the confounds of physiological noise in ALFF and enhance specificity and sensitivity in reflecting fMRI regional spontaneous activity by computing the ratio of the power spectrum of low-frequency to that of the entire frequency range.

#### ReHo

ReHo characterizes the so-called local functional connectivity (FC) between adjacent areas in a voxel-wise perspective. It measures the time series of a voxel and its nearest 26 neighbors and indexes the synchronization by Kendall’s coefficient of concordance (KCC)^52^. Some researchers have proposed local FC can alter the remote FC and affect the whole brain dynamics^57^.

#### Asymmetry index

In all analyses, asymmetry was expressed as the asymmetry index AI = (x_l _− x_r_)/(x_l_ + x_r_) with subscripts l and r identifying the left and right instances of value x, respectively (see for example^47,58,59^. Please note that AI is bounded by 1 (all activation in the left hemisphere) and − 1 (all activation in the right hemisphere) and that its directionality is completely arbitrary. In our formulation, AI becomes positive when x_l_ > x_r_ and negative when x_r_ > x_l_, thus a value > 0 means higher activation in the left hemisphere, and < 0 means higher activation in the right hemisphere. An increase in AI represents a shift toward left side activation and a decrease means a shift towards the right (applies throughout the manuscript).

#### Voxel-wise approach

For conducting voxel-wise asymmetry analyses, the individual parameter maps were mirrored at the midline (MNI x = 0), and the AI was calculated for every voxel from the original and flipped values. Second level group analyses were conducted on these images with one-sample and two-sample t-tests implemented in SPM GLMs with the covariates age and gender. A significance threshold of *p* < 0.05 cluster-level corr. with a cluster-defining threshold (CDT) of *p* = 0.001 unc. was applied in all imaging analyses. Because of the integration of lateralized information, our voxel-wise brain plots show results for both index directions (positive and negative) concurrently. Effects in a hemisphere reflect an increase in the respective voxels over the corresponding voxels in the other hemisphere.

#### Regional approach

**Figure 1:**
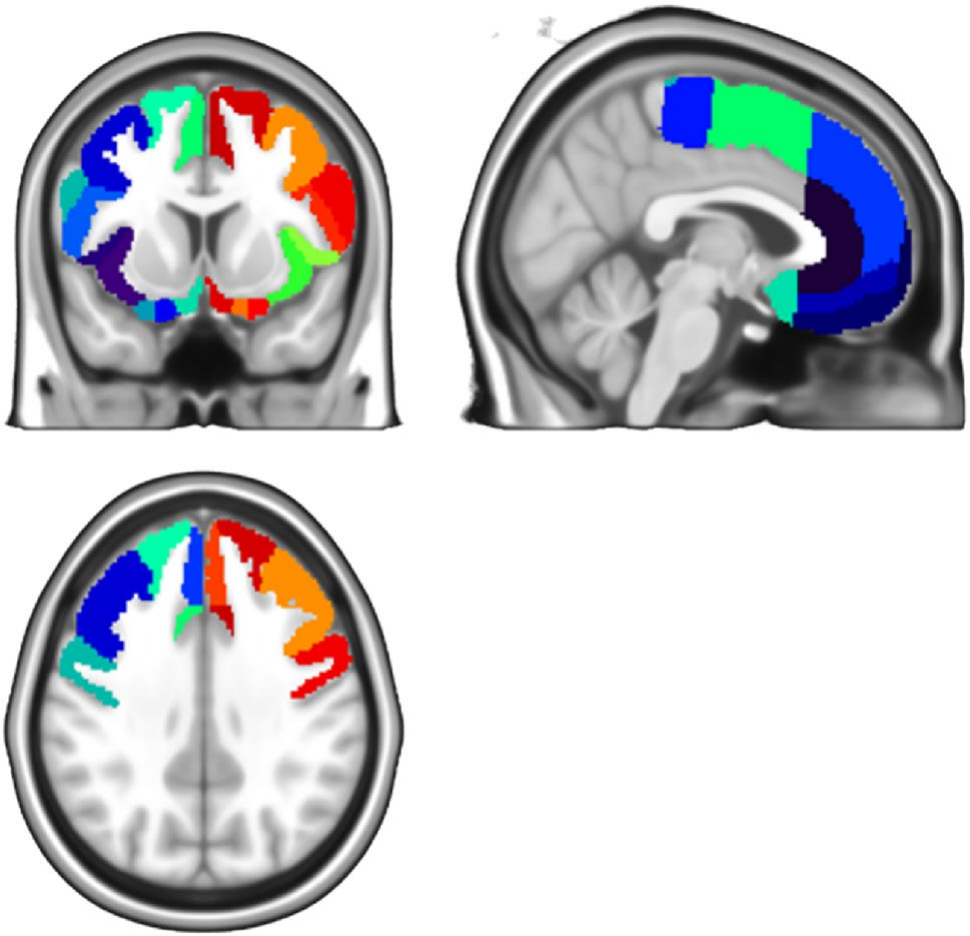
Regions in the regional approach. 20 parcels per hemisphere from the Neuromorphometrics atlas distributed with SPM in the frontal and anterior cingulate cortex were used for the regional analyses. Imaging parameters were averaged within the parcels and the asymmetry index for each corresponding pair was calculated. In addition, an average overall parcel was used to calculate asymmetry indices over the whole frontal hemisphere.

To apply another approach that is not dependent on the exact correspondence of specific voxels in the hemispheres and is able to capture potentially weaker distributed effects over larger parts of the cortex, we applied a second, regional analysis strategy based on anatomical parcellation. For this, we used the 20 Parcels per hemisphere of the Neuromorphometrics (NMM) atlas distributed with SPM that are covering the frontal and anterior cingulate cortex (Fig. 1; see region abbreviations in the supplement). Imaging parameters were averaged within the parcels and the asymmetry index for each corresponding parcel pair was calculated. In addition, the average overall parcels were used to calculate asymmetry indices over the whole frontal hemisphere. The p value threshold was corrected for multiple comparisons by Bonferroni correction, which is 0.0024 (0.05 divided by 21). The effect size (Hedge's g) and its confidence interval were reported and plotted in Figs. 3, 4 and 5. The 95% CI represents a 95% probability of containing the true parameter value (and its limits are equivalent to the significance threshold of a two-sided test with *p* < 0.05).

#### Levels of analyses

Our procedures allow us to conduct analyses at several different levels that are relevant to investigate associations of functional asymmetry with negative mood. Here, we report results from three levels: changes between rest and negative mood induction, group differences, and the relationship between changes in mood and changes in functional asymmetry values. (1) To investigate whether functional asymmetry changed over time, we analyzed the time difference between the rest phase and the induction phase per group with one sample t-tests. (2) To test the functional asymmetry differences across rMDD and healthy controls, we assessed group differences by two sample t-tests in the rest phase, the induction phase, and the change over time (induction—rest) respectively. (3) To test for the relationship between self-reported negative mood and functional asymmetry, we conducted correlation analyses within each group, during the rest phase, mood induction phase, and for the change over time (induction—rest) respectively. All analyses were corrected for age and gender. Similar analyses for positive mood scores are further reported in the supplement (Supplementary Table S1 and Figures S5, S6 & S7). As an extension to our analyses we conducted mixed-ANOVA models and report the significant results in Table 3 and the full results in Supplementary Table S3.

## Results

### Behavior

To validate the sad mood induction task, we compared the PANAS negative affect score before and after the task, and we compared it between groups (Table 1). After the sad mood induction task, both groups reported increased negative affect (rMDD: t(27) = 4.79, *p* < 0.001, g = 0.850; HC: t(27) = 3.88, *p* = 0.001, g = 0.688), and the two groups changed in varying degrees (t(56) = − 0.32, *p* = 0.002, g = − 0.848), that is, rMDD increased significantly more negative affect than HC. There is no significant group difference after the resting state, while rMDD and HC differed significantly after the induction task (rest state: t(56) = − 0.52, *p* = 0.609, g = − 0.131; induction state: t(56) = − 3.05, *p* = 0.004, g = − 0.778). Accordingly, the task was also able to reduce positive affect for both groups; see Supplementary Table S1 for detail.

**Table 1:** Demographic variables and PANAS negative score of both groups.

Variable	Group		p	t/c^2^	g	CI
	rMDD	HC				
n	30	30				
Gender (female/male)	20/10	21/9	0.781	0.08		
Age (years)	45.00 ± 7.90	44.53 ± 8.01	0.821	− 0.23		
*PANAS negative score*						
Rest phase	13.37 ± 3.30	12.87 ± 4.72	0.609	− 0.52	− 0.131	[− 0.640, 0.375]
Induction phase	20.17 ± 8.73	14.77 ± 5.59	0.004*	− 3.05	− 0.778	[− 1.313, 0.259]
Change (induction—rest)	6.80 ± 8.14	1.90 ± 2.62	0.002*	− 3.32	− 0.848	[− 1.387, − 0.325]
Time difference within rMDD^a^			< 0.001*	4.79	0.850	[0.443, 1.296]
Time difference within HC^a^			0.001*	3.88	0.688	[0.299, 1.107]

^a^Time difference is the difference between the rest phase and the induction phase per group.

*p < 0.05.

### Voxel-wise approach

In the voxel-wise analysis, we found very sparse results. We decided to report in the main text the results for the voxel-wise analyses which should theoretically show the strongest effects, namely the change in functional asymmetry over the experimental conditions in the rMDD group; and the association of the change in negative mood (PANAS negative score) with the change in functional asymmetry over the experimental conditions in the rMDD group. Full voxel-wise imaging results for all conducted analyses at a threshold of *p* = 0.001 unc. are reported in Supplementary Table S2.

**Figure 2:**
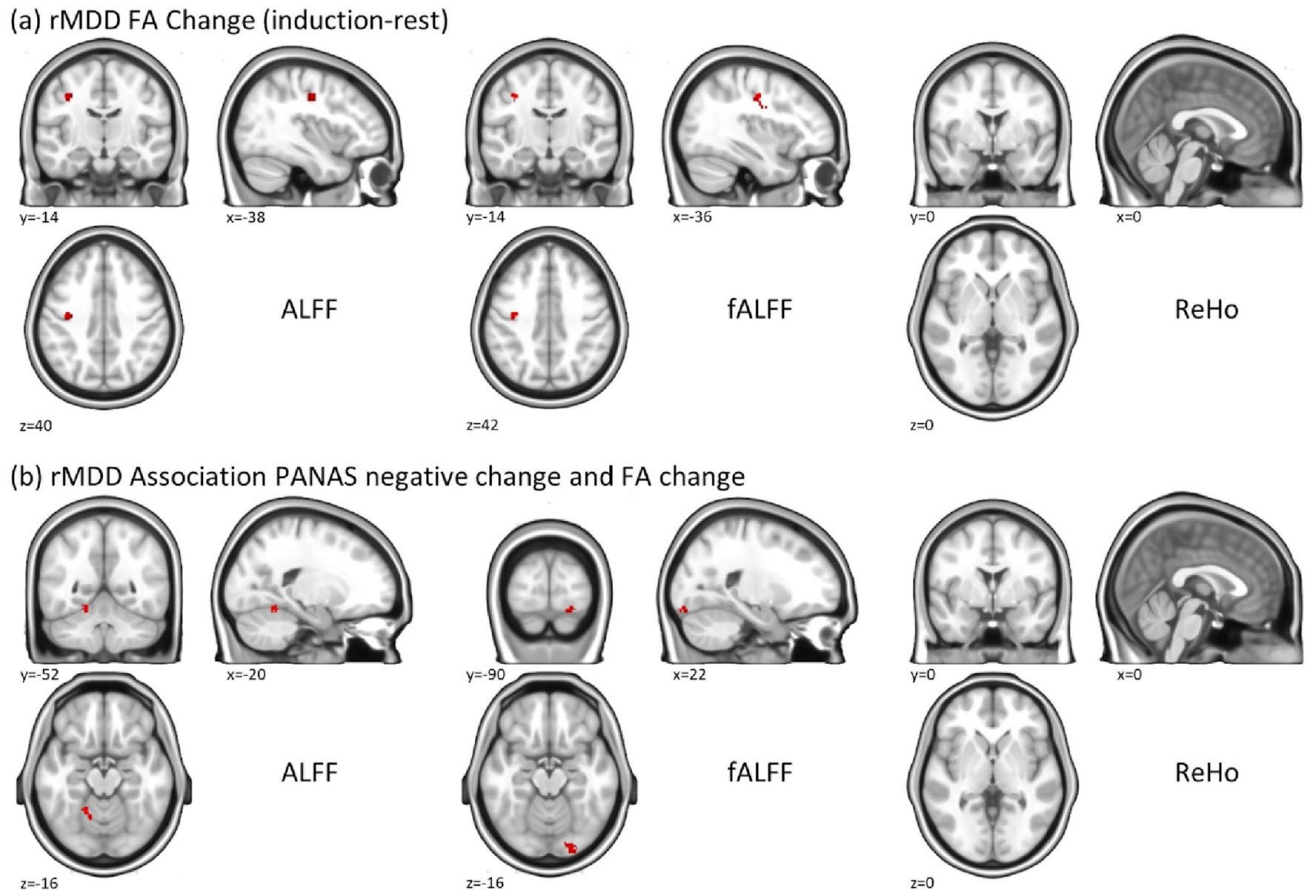
Results of the voxel-wise functional asymmetry analysis. Results are shown for the rMDD group for (**a**) the change in functional asymmetry between the rest and induction phase and (**b**) the association between the change in PANAS negative scores and the change in functional asymmetry. All maps are thresholded at a whole-brain cluster-level threshold of *p* = 0.05 with a cluster-defining threshold (CDT) of *p* = 0.001 unc. Please note that in the voxelwise analyses, a positive value in a hemisphere reflects an increase in the respective voxels over the corresponding voxels in the other hemisphere.

In the rMDD group, we identified a cluster in the left motor cortex with ALFF (k = 47 voxels) and fALFF (k = 48 voxels), but not with ReHo, which showed higher asymmetric activation in the induction phase compared to rest (Fig. 2a). In addition, we found a cluster (k = 48 voxels) in the left cerebellum with ALFF and a cluster in the right occipital fusiform gyrus (k = 42 voxels) with fALFF that showed an association between the change in the PANAS negative score and the change in functional asymmetry between the rest and induction phase in the rMDD group. Again, no effect was significant in the ReHo analyses (Fig. 2b). Significant results are summarized in Table 2.

**Table 2:** Significant results of the voxel-wise functional asymmetry analysis.

	p_FWE-corr_	Cluster size	Peak t value	MNI		
				x	y	z
ALFF change of rMDD^a^	0.031*	47	7.13	− 38	− 14	40
fALFF change of rMDD^b^	0.011*	48	4.99	− 36	− 14	42
			4.08	− 36	− 6	30
PANAS negative change correlates to ALFF change of rMDD^c^	0.026*	48	4.39	− 20	− 52	− 16
			4.10	− 14	− 58	− 14
PANAS negative change correlates to fALFF change of rMDD^d^	0.025*	42	4.60	22	− 90	− 16

^a^ALFF functional asymmetry has higher asymmetric activation in the induction phase compared to rest.

^b^fALFF functional asymmetry has higher asymmetric activation in the induction phase compared to rest.

^c^The change in the PANAS negative score correlates to the change in ALFF functional asymmetry.

^d^The change in the PANAS negative score correlates to the change in fALFF functional asymmetry. Change is defined as induction—rest.

*p < 0.05.

### Regional approach

Regional functional asymmetry changes between the rest phase and negative mood induction phase.

**Figure 3:**
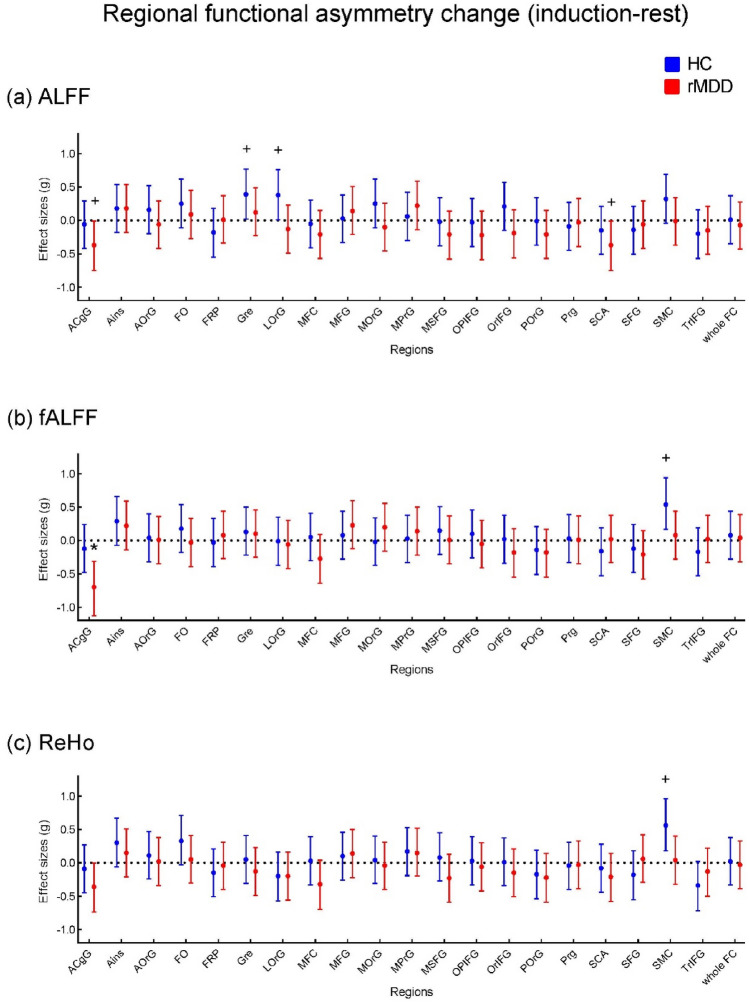
Regional functional asymmetry change between the induction phase and the rest phase. Dots represent Hedge's g, the error bar represents 95% confidence interval. The effect size of the time difference between the induction phase and the rest phase was estimated based on the t-value. A positive effect size reflects that functional asymmetry in the induction phase is higher than in the resting state. See the supplement for region abbreviations, regions with significant results are described above in the text. ^+^*p* < 0.05, *p < 0.0024.

We defined the functional asymmetry change as the difference of functional asymmetry between the rest phase and the negative mood induction phase (induction—rest). A positive change reflects that functional asymmetry in the induction phase is higher than that in the resting state, that is, functional asymmetry increased after the sad mood induction task, and a negative change reflects a decreased functional asymmetry. We depicted the functional asymmetry changes (ALFF, fALFF, and ReHo respectively) of the 21 regions per group in Fig. 3. At the nominal significance level, the rMDD group showed decreased ALFF functional asymmetry in ACgG (anterior cingulate gyrus) (t(27) = − 2.10, *p* = 0.046, g = − 0.372) and SCA (subcallosal area) (t(27) = − 2.08, *p* = 0.047, g = − 0.370), and decreased fALFF functional asymmetry in ACgG (t(27) = − 3.97, *p* = 0.00048, g = − 0.704) after the sad mood induction task; while the HC group showed increased ALFF functional asymmetry in GRe (gyrus rectus) (t(27) = 2.17, *p* = 0.039, g = 0.386) and LOrG (lateral orbital gyrus) (t(27) = 2.14, *p* = 0.042, g = 0.380), increased fALFF functional asymmetry in SMC (supplementary motor cortex) (t(27) = 3.07, *p* = 0.005, g = 0.545), and increased ReHo functional asymmetry in SMC (t(27) = 3.17, *p* = 0.004, g = 0.563) (Fig. 3). Please find functional asymmetry values of the 21 regions in the rest state and induction state respectively in Supplementary Figure S1 and Figure S2.

The decrease in fALFF functional asymmetry in the ACgG (anterior cingulate gyrus) of rMDD was the only effect in our main regional analyses that reached a significance threshold that was Bonferroni corrected for the number of regions (*p* < 0.0024). Interestingly, the effect is based on an elevated functional asymmetry in the rMDD in the baseline resting state that returns exactly to the level of the HC group in the induction phase (see Supplementary Figure S8).

#### Group differences

**Figure 4:**
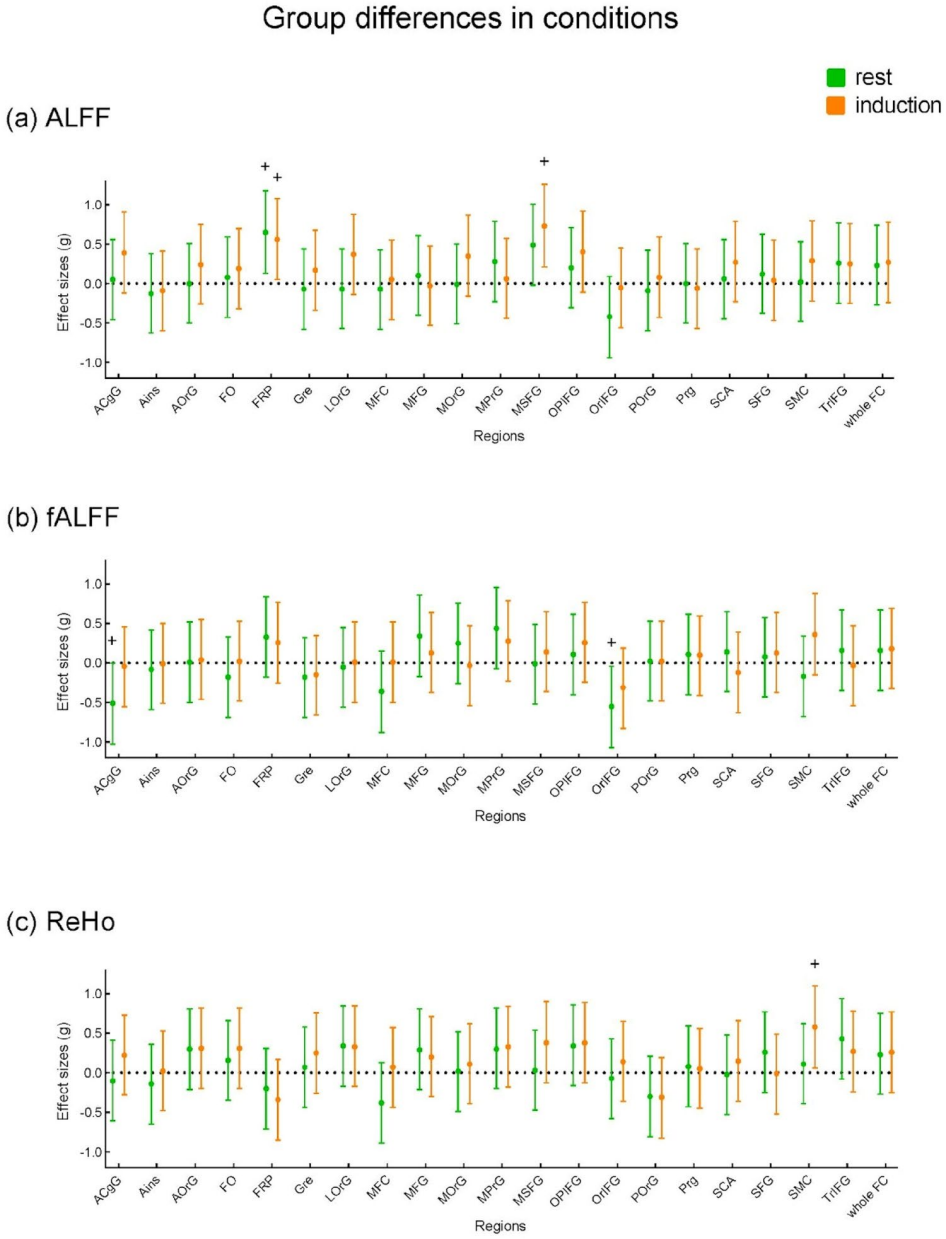
Group differences of the regional functional asymmetry between rMDD and HC in the rest and induction phase. Dots represent Hedge's g, the error bar represents 95% confidence interval. The effect size of the group difference was estimated based on the t-value. A positive effect size reflects that rMDD has lower functional asymmetry than HC. See the supplement for region abbreviations, regions with significant results are described above in the text. + *p* < 0.05.

**Figure 5:**
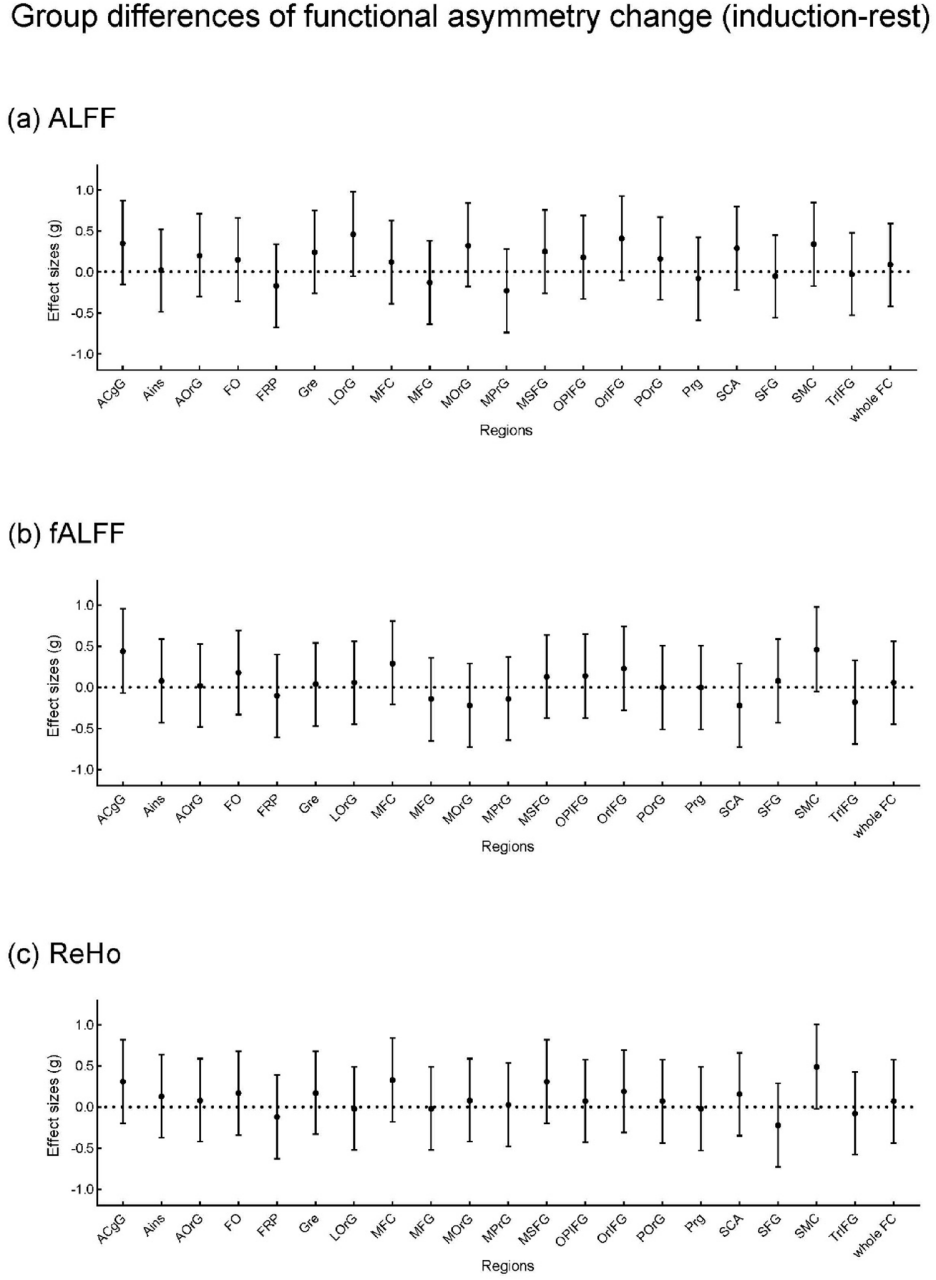
Group differences of the regional functional asymmetry change (induction—rest) between rMDD and HC. Dots represent Hedge's g, the error bar represents 95% confidence interval. The effect size of the group difference was estimated based on the t-value. A positive effect size reflects that rMDD has lower functional asymmetry change than HC. No significant results were identified. See the supplement for region abbreviations.

We compared functional asymmetry between the rMDD group and the HC group in the rest state and the induction phase (Fig. 4), and the change over time (Fig. 5) respectively. In the resting state, compared to HC, rMDD showed lower ALFF functional asymmetry in FRP (frontal pole) (t(56) = 2.55, *p* = 0.014, g = 0.650), higher fALFF functional asymmetry in ACgG (anterior cingulate gyrus) (t(56) = − 2.01, *p* = 0.049, g = − 0.513) and OrlFG (orbital part inferior frontal gyrus ) (t(56) = − 2.15, *p* = 0.036, g = − 0.548). In the induction state, rMDD showed lower ALFF functional asymmetry in FRP (frontal pole) (t(56) = 2.19, *p* = 0.032, g = 0.560) and MSFG (superior frontal gyrus medial ) (t(56) = 2.85, *p* = 0.006, g = 0.728), and lower ReHo functional asymmetry in SMC (supplementary motor cortex) (t(56) = 2.26, *p* = 0.028, g = 0.577). No nominally significant group differences were identified in the functional asymmetry change (induction-rest).

An effect that might be of potential interest but is contrary to an expected stronger change in the rMDD group is the nominal group difference in the SMC (supplementary motor cortex) for ReHo functional asymmetry change. This difference is based on an increase in fALFF functional asymmetry and ReHo functional asymmetry in the HC group while these values are not changed in the rMDD group (see supplementary Figures S9 & S10).

We also conducted mixed-model ANOVA as an extension to our analyses, and the results were consistent with the results reported above. There were significant main effects on several ROIs, but the 2 (group: rMDD or HC) × 2 (time: rest or induction) mix ANOVA revealed no significant group-by-time interactions. (see Table 3 for significant results and supplementary Table S3 for full results).

**Table 3:** Significant Results of Mixed-model ANOVA.

Variable/ROI	Group effect		Time effect		Group * time	
	F	p	F	p	F	p
PANAS—negative	5.025	0.029^+^	31.040	< 0.001*	9.846	0.003^+^
PANAS—positive	2.595	0.113	10.653	0.002*	2.451	0.123
ALFF:						
FRP	7.195	0.010^+^	0.188	0.666	0.311	0.579
GRe	0.069	0.794	4.142	0.046^+^	0.982	0.326
MSFG	5.913	0.018^+^	1.155	0.287	0.891	0.349
SCA	0.618	0.435	4.681	0.035^+^	1.425	0.238
fALFF:						
ACgG	2.201	0.143	8.224	0.006^+^	3.365	0.072
SMC	0.153	0.697	6.091	0.017^+^	3.414	0.070
ReHo:						
SMC	2.207	0.143	4.707	0.034^+^	3.381	0.071

See the supplement for region abbreviations. ^+^*p* < 0.05, *p < 0.0024.

#### Relationship between changes in negative mood and changes in functional asymmetry values

**Figure 6:**
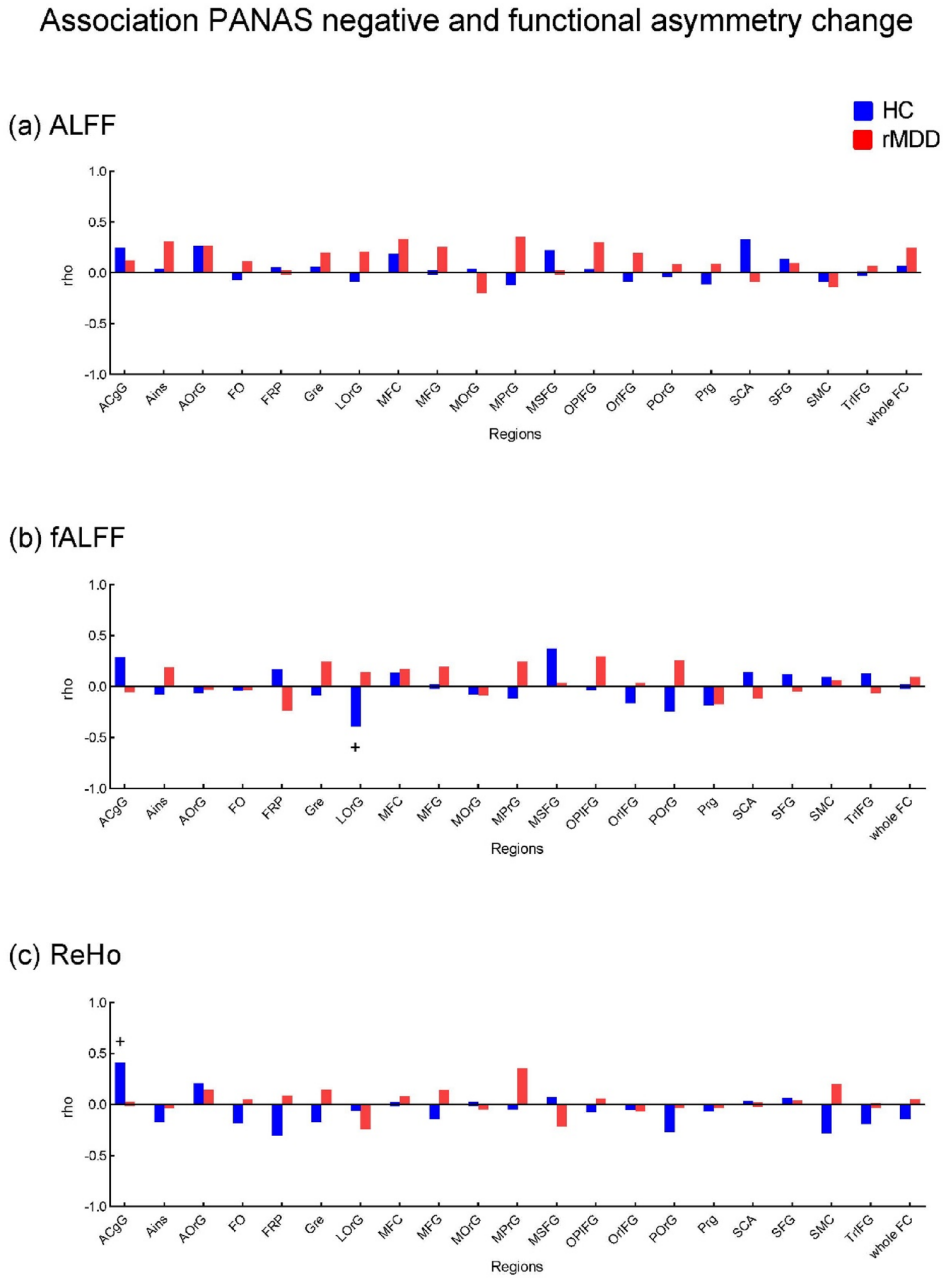
Relationship between the change in PANAS negative score and the change in functional asymmetry. The change is defined as Induction—Rest. The bar chart presents the strength of the association (rho). See the supplement for region abbreviations, regions with significant results are described above in the text. ^+^*p* < 0.05.

To capture the association between the negative mood and the functional asymmetry, we calculated the partial correlation between the PANAS negative change (induction-rest) and the functional asymmetry change (induction-rest) corrected for age and gender (Fig. 6). No significant associations were identified in the rMDD group. In HC, PANAS negative change has a negative correlation with fALFF functional asymmetry change in LOrG (lateral orbital gyrus) (rho = − 0.39, *p* = 0.041); and has a positive correlation with ReHo functional asymmetry change in ACgG (anterior cingulate gyrus) (rho = 0.41, *p* = 0.030). Please find the results for associations between PANAS score and functional asymmetry during the resting state and the induction phase respectively in the supplementary Figure S3 & S4.

Overall, the results we obtained are very limited and do not contain a clearly identifiable pattern that corresponds to expected effects if an underlying relationship between functional asymmetry values and negative mood would be present.

## Discussion

In this study, we reanalyzed an existing fMRI data set^48^ on negative mood induction in participants with rMDD and HC and addressed the question of whether evidence for functional asymmetry of frontal brain regions can be found in the data. We were specifically interested in this question because of our aim to explore potential target processes for real-time fMRI neurofeedback interventions. With this analysis, we wanted to test whether a negative-mood related functional asymmetry effect, which might be a promising neurofeedback candidate process, can be identified in fMRI. While FA effects have been extensively studied especially with EEG, the fMRI literature on this topic is rather limited^37,40,60–64^.

After extensively exploring our results, we came to the conclusion that we were not able to detect FA effects related to negative emotion with our fMRI analyses. While, as expected by chance, some individual tests showed nominal significance, the results as a whole are probably best characterized as a ‘dance of p-values’ around a zero effect and lack any clearly interpretable congruent and systematic patterns. Even the results we found at nominal significance levels turned out to be based on underlying changes and group differences that were not corresponding to patterns expected if the expected effects would really be present.

It seems important to emphasize that this failure to detect interpretable results was not our intention in this study. We conducted our analyses with the goal to obtain positive results, and were aiming to implement straightforward and meaningful analysis strategies. Although we did not pre-register the analysis plan, which retrospectively would have been preferable, we think that our procedures are comprehensible and well justified.

While the research question was not planned when the original study was set up, this study was designed in an almost optimal way to address our research question. If we would have designed a new study we would have come up with a virtually identical design. The experimental task clearly evoked negative mood in the participants (Table 1), and the study design allows to investigate the different intra- and interindividual effects that should be present if FA effects are present and detectable with fMRI. Also, while the analyzed data set is not especially large, and our analyses might therefore not have the highest power, the data set still has a decent size and is of high quality, which could for example be seen by the good matching of the rMDD and HC group.

Another limitation of our study is the use of a normalization procedure based on the functional images in the re-analyzed data set, which potentially reduces the spatial precision of the voxel-wise analyses. To alleviate these spatial precision and power problems, we conducted not only voxel-wise but also regional analyses, which can be expected to be more sensitive, and report whole-brain results for every analysis thresholded at *p* < 0.001 uncorrected in the supplement.

We used three different measures to assess fMRI activation, namely ALFF, fALFF, and ReHo. All of these measures were originally developed for resting state analyses, but are very well suited to analyze our mood induction data, which has very little task structure and mostly, on the temporal level, resembles a resting state measurement. The induced negative mood can be assumed to be present over the whole experimental phase, and related effects should thus theoretically be detectable by our procedures.

Obviously, we used a specific analysis pipeline on already existing data, and, given the high flexibility of fMRI analyses, other analysis strategies are possible, which might have yielded somewhat different results if they were used. It was neither feasible for us nor our goal to explore all possible analysis strategies in this single study. However, if a FA effect plays a major role in negative mood and can be detected by fMRI we would expect it to be reflected in our results at least in some of our complex analyses.

Interestingly, the putative inability of fMRI to detect FA effects in comparison to EEG has already been recognized. Kelley et al.^65^ have discussed this topic and suggested that possible reasons are probably the supine position, which has been demonstrated can reduce approach motivation and decreases left frontal asymmetry^66^; and their different physiological basis, as fMRI indirectly measures the neuronal activity with BOLD response and the hemodynamic response in fMRI measurement is dependent on action potentials and cortical stellate cells. Morys et al.^47^ further discussed this incongruence may be due to alpha power and fALFF measuring different processes, and fMRI measures a different frequency range of oscillations than EEG (0.01–0.1 Hz vs. 8–12 Hz). Another possible reason could arise from the validation of the FA theory: although there is meta-analysis supporting the theory in affective disorders that depression is related to left side frontal asymmetry^67^, not all studies observed this link (e.g.^36,68,69^), and a recent meta-analysis review did not find significant results but only suggested a slight tendency toward left lateralization in the depression group^70^. Another meta-analysis also reported non-significant results when exploring the diagnostic value of frontal asymmetry in depression^27^. Similarly, Kołodziej et al. ^71^ concluded in their meta anaylsis that treating EEG frontal alpha asymmetry as a biomarker of depressive disorders is not sufficiently empirically grounded as only 13 of their 270 analyses revealed significant results.

Especially in this situation, it seems relevant that negative findings as ours are published so that other researchers addressing similar questions get a full picture of the state of knowledge. We suspect that the relative lack of studies investigating functional asymmetry effects with fMRI might hint to a file drawer problem and that probably studies like ours were conducted but also yielded negative results and were not published.

A limitation of our study is that we only investigated participants with remitted MDD. While this group clearly responded more strongly to the negative mood induction than the HC group, we can not exclude that functional asymmetry effects could play a specific role in acute MDD, although we consider this unlikely, given the assumed trait character of frontal asymmetry effects. Moreover, the limited sample size in our study in combination with a small effect size of functional asymmetry changes may have contributed to our non-significant results. However, since an effect must be observable in a rather small sample to be a reasonable target for NFB, which is conducted on the single individual level, our results suggest that FA is likely not a plausible candidate for fMRI NFB approaches. It would not make any sense to train a very small effect or an effect that can only be observed in a small subgroup of affected individuals. While we think that our results are not providing any evidence for the presence of a FA effect associated with negative mood induction in fMRI, other researchers might interpret specific findings in our results differently. If this would be the case, our standardized effect sizes for all regional analyses could be used for planning the sample size of future studies investigating these effects.

From our understanding of our results, we conclude that it seems not advisable to consider FA as a target for real-time fMRI neurofeedback interventions for mood disorders, as we were not able to systematically establish the expected underlying phenotype. In EEG NFB, frontal alpha asymmetry has been tested as a targeted intervention approach for depressive patients (see for example^29^) and several of these investigations have reported clinically relevant improvements. However, most NFB studies published so far were either non-randomized, non-blinded, or single-armed. High quality experimental designs, as suggested by the CRED-nf checklist^72^, are urgently needed to systematically examine frontal alpha asymmetry EEG NFB approaches and testing the presence of the suspected mechanisms.

### Supplementary Information

Supplementary Information.

## Data Availability

The dataset generated and analysed during the current study are not publicly available due to data protection laws but are available from the corresponding author on reasonable request.
