# Supplementary material for: No evidence from a negative mood induction fMRI task for frontal functional asymmetry as a suitable neurofeedback target
Source: Sci Rep. 2023 Oct 16;13:17557. doi: 10.1038/s41598-023-44694-3 (PMC10579342; doi:10.1038/s41598-023-44694-3)
Supplement: Supplementary file 1 — Supplementary Information. [file 41598_2023_44694_MOESM1_ESM.docx]

**Supplement**

**Region Abbreviations**

1 - ACgG: anterior cingulate gyrus (r: 100; l:101)

2 - AIns: anterior insula (r:102 ; l: 103)

3 - AOrG: anterior orbital gyrus (r: 104 ; l: 105)

4 - FO: frontal operculum (r: 118; l: 119 )

5 - FRP: frontal pole (r: 120; l: 121 )

6 - GRe: gyrus rectus (r: 124; l: 125)

7 - LOrG: lateral orbital gyrus (r: 136; l: 137 )

8 - MFC: medial frontal cortex (r: 140; l: 141)

9 - MFG: middle frontal gyrus (r: 142 ; l: 143 )

10 - MOrG: medial orbital gyrus (r: 146; l: 147)

11 - MPrG: precentral gyrus medial (r: 150; l: 151)

12 - MSFG: superior frontal gyrus medial (r: 152; l:153 )

13 - OPIFG: opercular part inferior frontal gyrus (r: 162; l: 163 )

14 - OrIFG: orbital part inferior frontal gyrus (r: 164; l: 165)

15 - POrG: posterior orbital gyrus (r: 178; l: 179 )

16 - Prg: precentral gyrus (r: 182; l:183 )

17 - SCA: subcallosal area (r:186 ; l: 187 )

18 - SFG: superior frontal gyrus (r: 190; l: 191 )

19 - SMC: supplementary motor cortex (r: 192; l: 193)

20 - TrIFG: triangular part inferior frontal gyrus (r: 204; l: 205)

21 – whole frontal cortex (all of the above)

**Table S1. Group Differences and Time Differences of PANAS Positive Score**

| variable | group | | p | t/χ^2^ | g | CI |
| --- | --- | --- | --- | --- | --- | --- |
|  | rMDD | HC |  |  |  |  |
| rest | 26.87±6.37 | 28.60±6.61 | 0.309 | 1.03 | 0.262 | [-0.245,0.773] |
| induction | 23.83±6.45 | 27.53±8.27 | 0.046* | 2.04 | 0.519 | [0.008,1.040] |
| change (induction - rest) | -3.03±5.75 | -1.07±3.79 | 0.082 | 1.77 | 0.452 | [-0.057,0.970] |
| time difference within rMDD^a^ | |  | 0.006* | -3.00 | -0.533 | [-0.931,-0.158] |
| time difference within HC^a^ | |  | 0.115 | -1.63 | -0.289 | [-0.661,0.072] |

a: time difference is the difference between the rest phase and the induction phase per group; *p＜0.05

**Table S2. Full Imaging Results of the Voxel-Wise Functional Asymmetry Analysis**

| 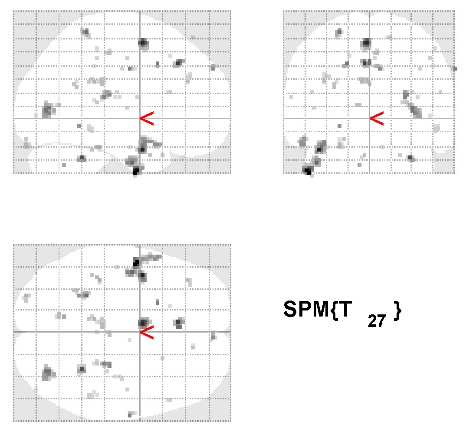 | 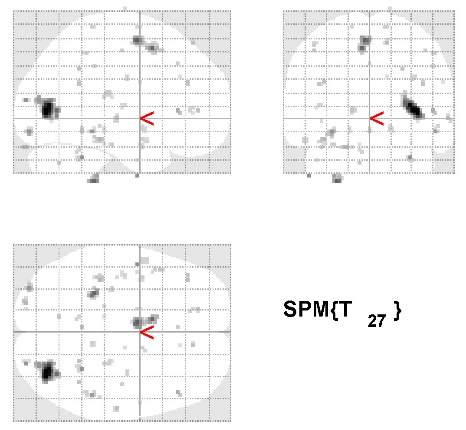 | 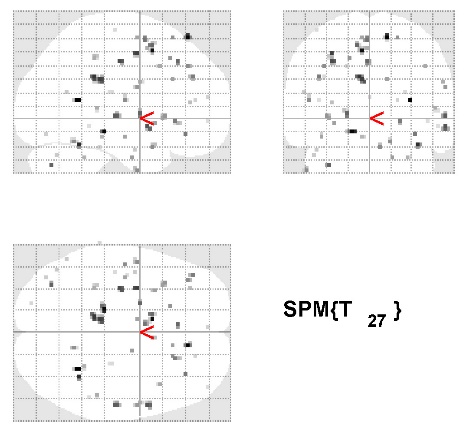 |
| --- | --- | --- |
| Time differences of ALFF-functional asymmetry change in HC | Time differences of fALFF- functional asymmetry change in HC | Time differences of ReHo- functional asymmetry change in HC |
| cluster-level: p_fwe_= 0.003, K_E_= 64.  peak-level: p_fwe_= 0.958, T= 5.51, MNI=-52 -4 -42.  p_fwe_= 1.000, T= 4.49, MNI= -44 -8 -36.  cluster-level: p_fwe_= 0.040, K_E_= 43.  peak-level: p_fwe_= 1.000, T= 5.07, MNI=-4 0 56. | cluster-level: p_fwe_= 0.000, K_E_= 173.  peak-level: p_fwe_= 0.102, T= 6.59, MNI=32 -76 4.  p_fwe_= 1.000, T= 5.01, MNI= 34 -68 4.  p_fwe_= 1.000, T= 4.37, MNI= 28 -82 12.  cluster-level: p_fwe_= 0.001, K_E_= 68.  peak-level: p_fwe_= 1.000, T= 5.16, MNI= -6 -4 58.  p_fwe_= 1.000, T= 5.04, MNI= -8 8 52. |  |
| 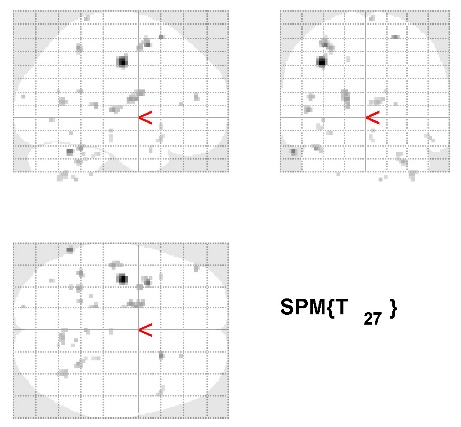 | 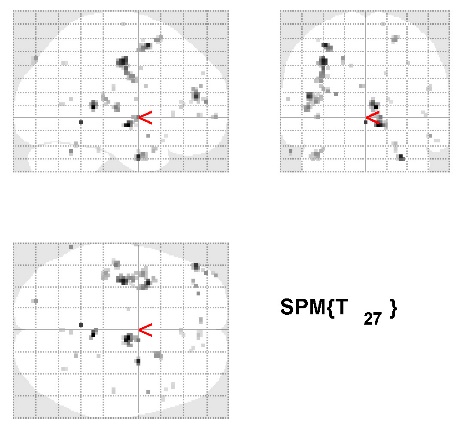 | 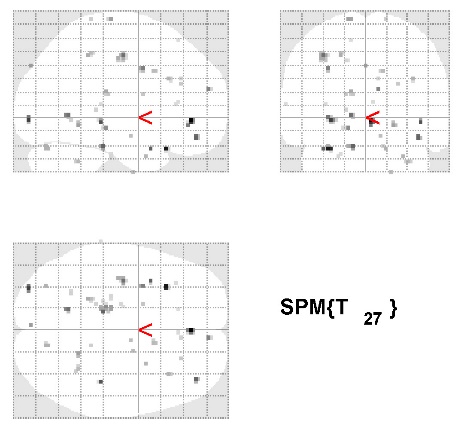 |
| Time differences of ALFF- functional asymmetry change in rMDD | Time differences of fALFF- functional asymmetry change in rMDD | Time differences of ReHo- functional asymmetry change in rMDD |
| cluster-level: p_fwe_= 0.031, K_E_= 47.  peak-level: p_fwe_= 0.026, T= 7.13, MNI=-38 -14 40. | cluster-level: p_fwe_= 0.011, K_E_= 48.  peak-level: p_fwe_= 1.000, T= 4.99, MNI=-36 -14 42.  p_fwe_= 1.000, T= 4.08, MNI=-36 -6 30. |  |
| 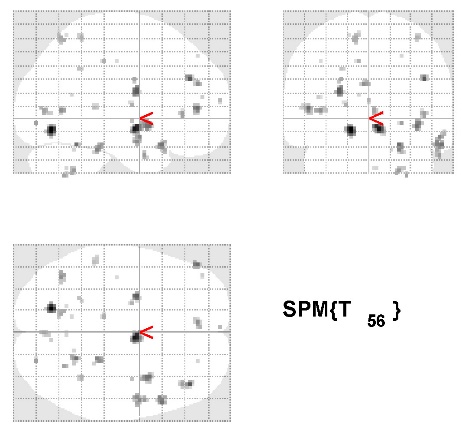 | 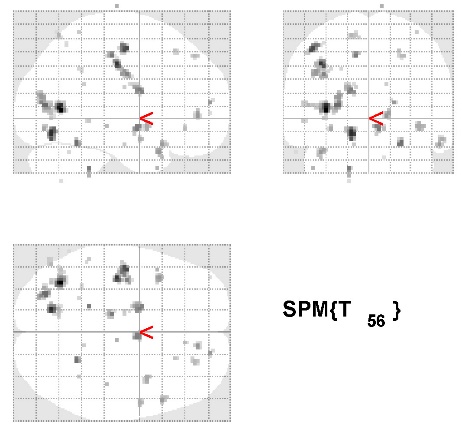 | 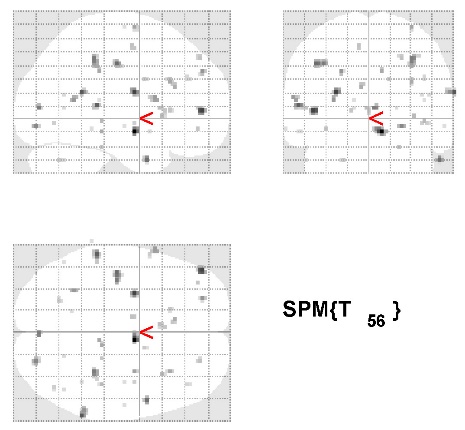 |
| Group differences of ALFF- functional asymmetry rest | Group differences of fALFF- functional asymmetry rest | Group differences of ReHo- functional asymmetry rest |
|  | cluster-level: p_fwe_=0.004, K_E_= 70.  peak-level: p_fwe_= 0.630, T= 5.20, MNI= -36 -64 4.  p_fwe_= 1.000, T= 3.54, MNI= -36 -72 2.  p_fwe_= 1.000, T= 3.50, MNI= -46 -64 8.  cluster-level: p_fwe_=0.022, K_E_= 54.  peak-level: p_fwe_= 0.993, T= 4.66, MNI= -40 -20 40.  p_fwe_= 1.000, T= 4.03, MNI= -38 -12 32. |  |
| 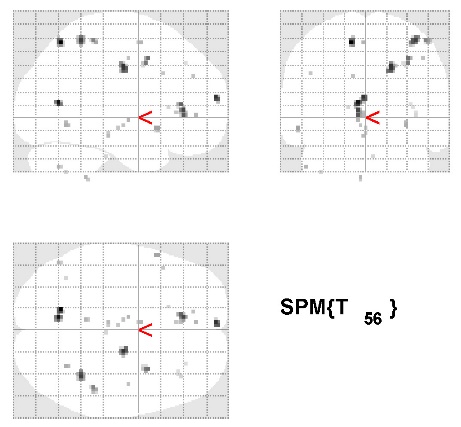 | 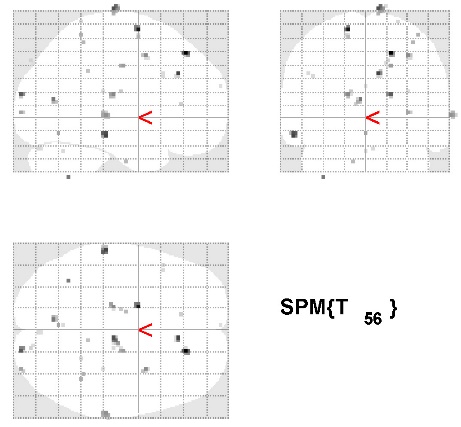 | 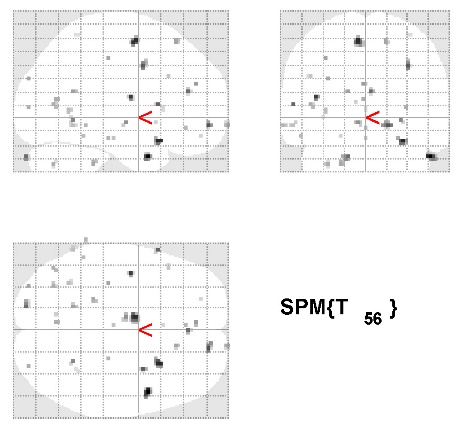 |
| Group differences of ALFF- functional asymmetry induction | Group differences of fALFF- functional asymmetry induction | Group differences of ReHo- functional asymmetry induction |
|  |  |  |
| 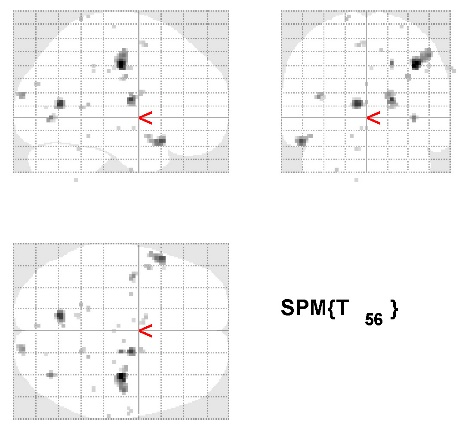 | 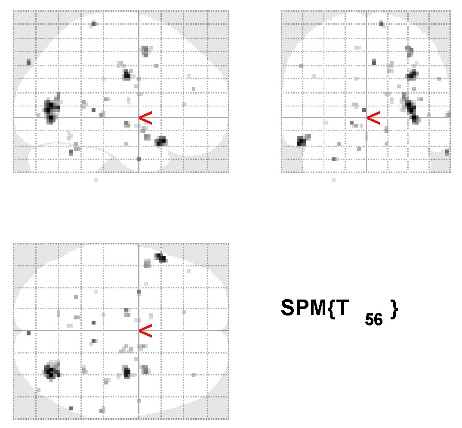 | 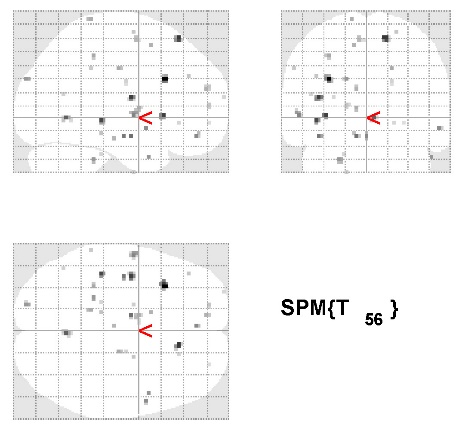 |
| Group differences of ALFF- functional asymmetry change | Group differences of fALFF- functional asymmetry change | Group differences of ReHo- functional asymmetry change |
| cluster-level: p_fwe_= 0.001, K_E_= 89.  peak-level: p_fwe_= 0.671, T= 5.15, MNI= 38 -16 40.  p_fwe_= 1.000, T= 4.13, MNI= 48 -14 46. | cluster-level: p_fwe_=0.000, K_E_= 110.  peak-level: p_fwe_= 0.994, T= 4.69, MNI= 36 -72 -2.  p_fwe_= 0.997, T= 4.64, MNI= 32 -72 8.  p_fwe_= 1.000, T= 3.81, MNI= 28 -66 14. |  |
| 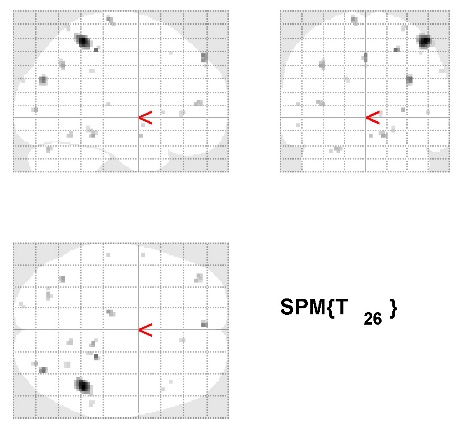 | 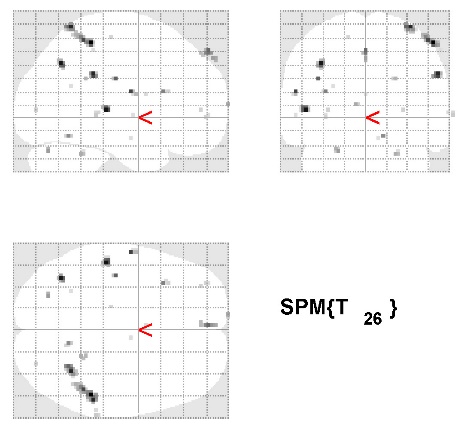 | 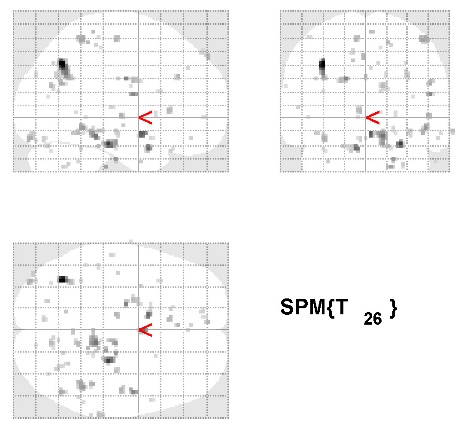 |
| Association PANAS negative and ALFF-functional asymmetry rest in HC | Association PANAS negative and fALFF-functional asymmetry rest in HC | Association PANAS negative and ReHo-functional asymmetry rest in HC |
| cluster-level: p_fwe_= 0.005, K_E_= 94.  peak-level: p_fwe_= 0.991, T= 5.12, MNI= 46 -46 58. | cluster-level: p_fwe_= 0.047, K_E_= 42.  peak-level: p_fwe_= 1.000, T= 5.09, MNI= 52 -40 56.  p_fwe_= 1.000, T= 4.08, MNI= 46 -48 60. |  |
| 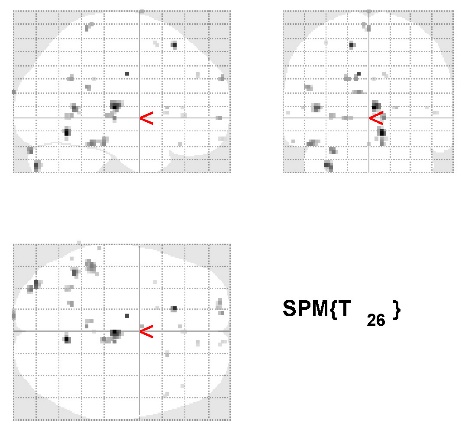 | 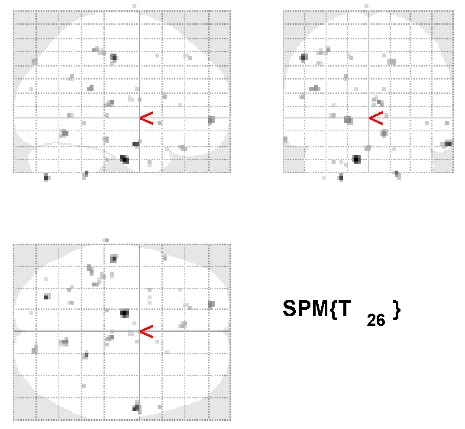 | 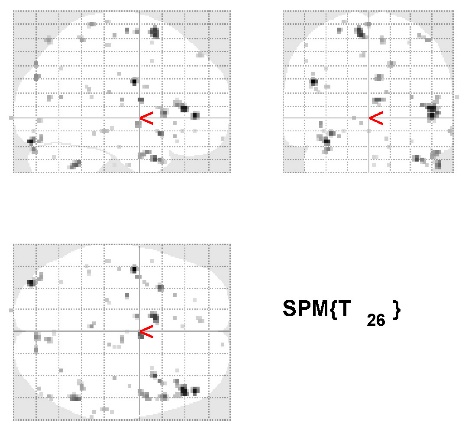 |
| Association PANAS negative and ALFF- functional asymmetry induction in HC | Association PANAS negative and fALFF- functional asymmetry induction in HC | Association PANAS negative and ReHo- functional asymmetry induction in HC |
|  |  |  |
| 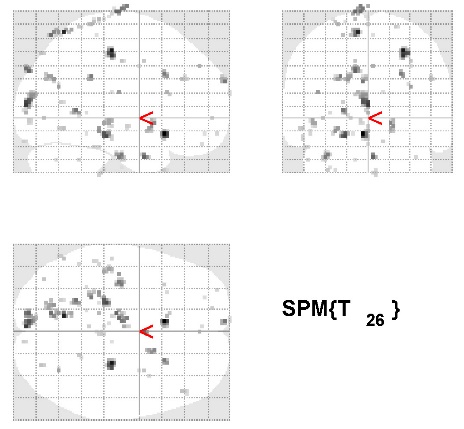 | 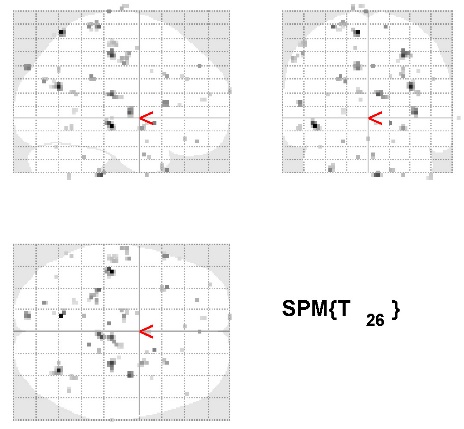 | 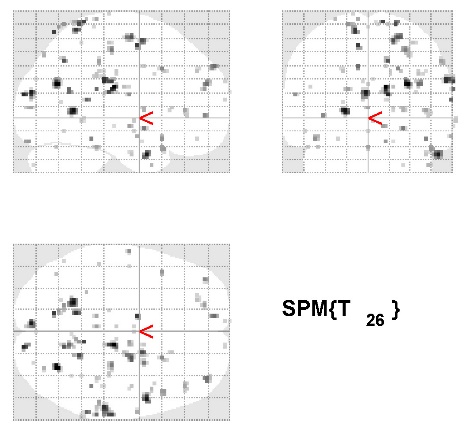 |
| Association PANAS negative and ALFF- functional asymmetry change in HC | Association PANAS negative and fALFF-functional asymmetry change in HC | Association PANAS negative and ReHo- functional asymmetry change in HC |
|  |  |  |
| 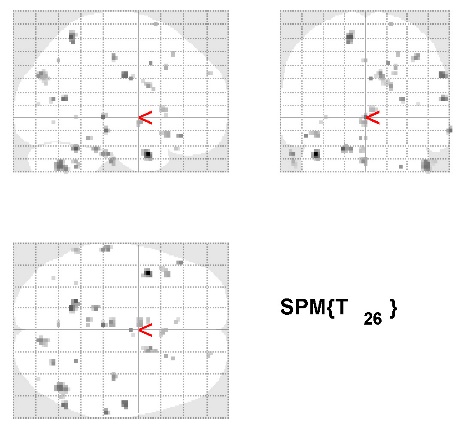 | 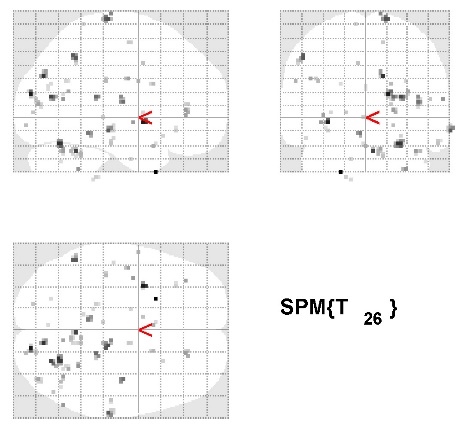 | 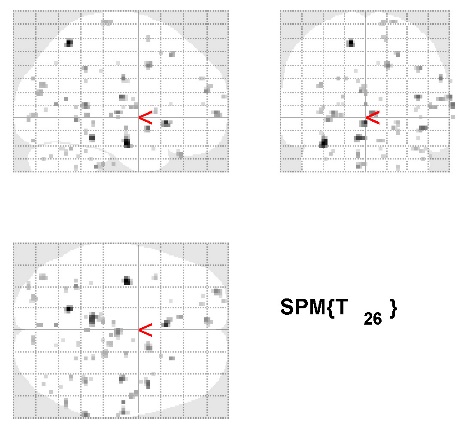 |
| Association PANAS positive and ALFF- functional asymmetry rest in HC | Association PANAS positive and fALFF- functional asymmetry rest in HC | Association PANAS positive and ReHo- functional asymmetry rest in HC |
|  |  |  |
| 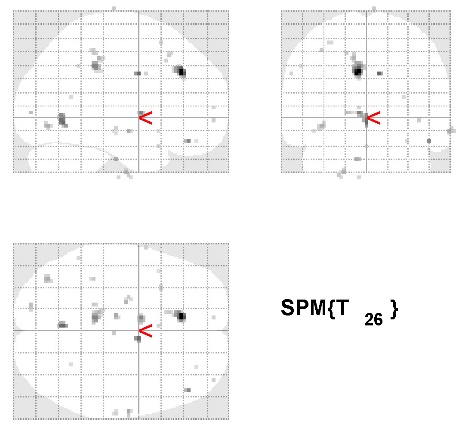 | 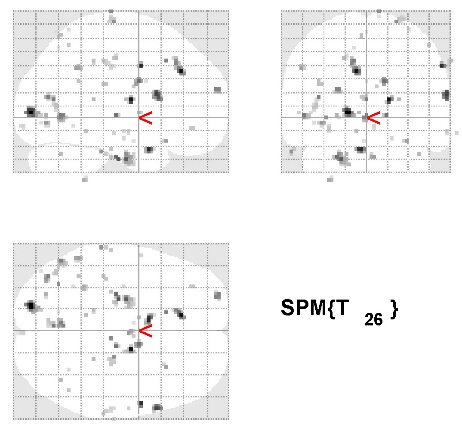 | 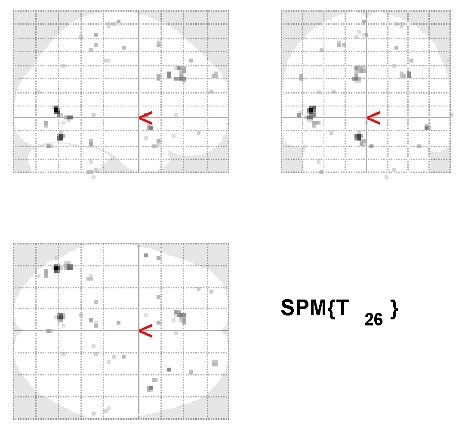 |
| Association PANAS positive and ALFF- functional asymmetry induction in HC | Association PANAS positive and fALFF- functional asymmetry induction in HC | Association PANAS positive and ReHo- functional asymmetry induction in HC |
|  |  |  |
| 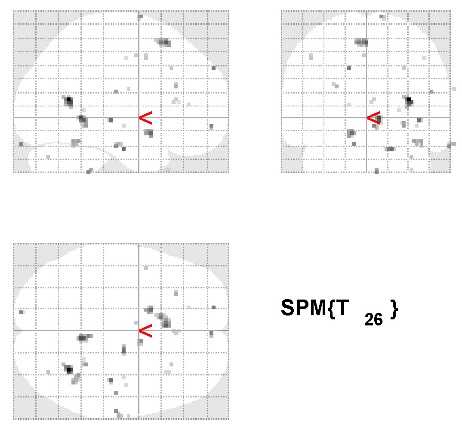 | 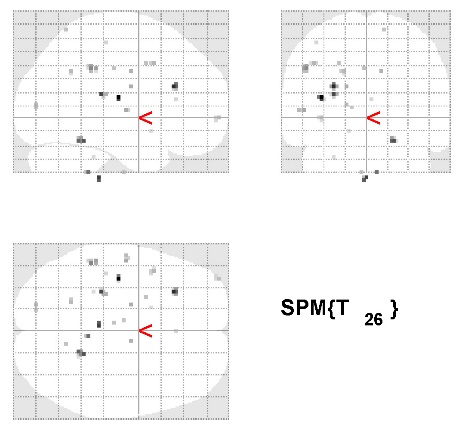 | 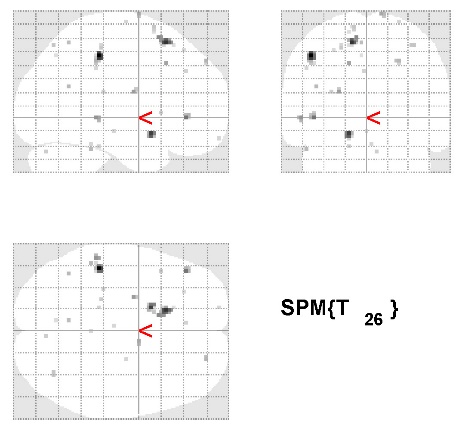 |
| Association PANAS positive and ALFF- functional asymmetry change in HC | Association PANAS positive and fALFF- functional asymmetry change in HC | Association PANAS positive and ReHo- functional asymmetry change in HC |
|  |  |  |
| 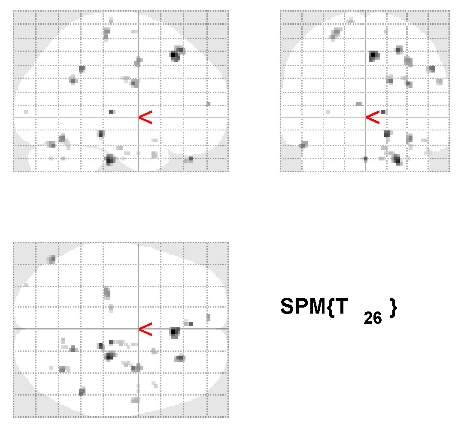 | 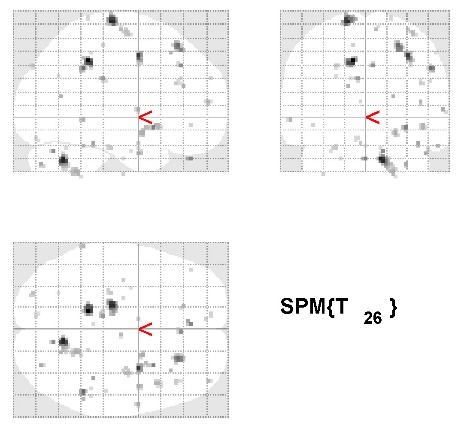 | 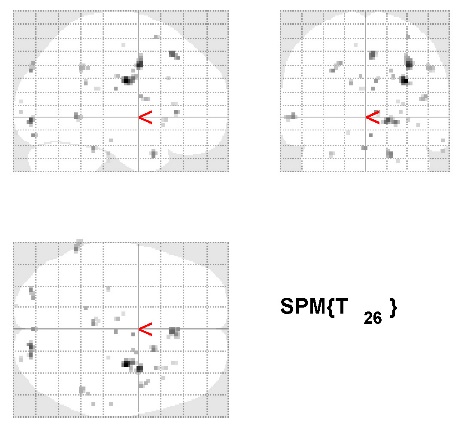 |
| Association PANAS negative and ALFF- functional asymmetry rest in rMDD | Association PANAS negative and fALFF-functional asymmetry rest in rMDD | Association PANAS negative and ReHo-functional asymmetry rest in rMDD |
|  | cluster-level: p_fwe_= 0.037, K_E_= 44.  peak-level: p_fwe_= 0.992, T= 5.35, MNI= 10 -62 -34.  cluster-level: p_fwe_= 0.020, K_E_= 49.  peak-level: p_fwe_= 0.996, T= 5.29, MNI= -16 -22 72. |  |
| 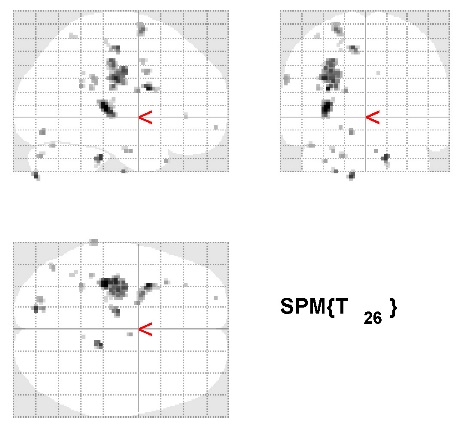 | 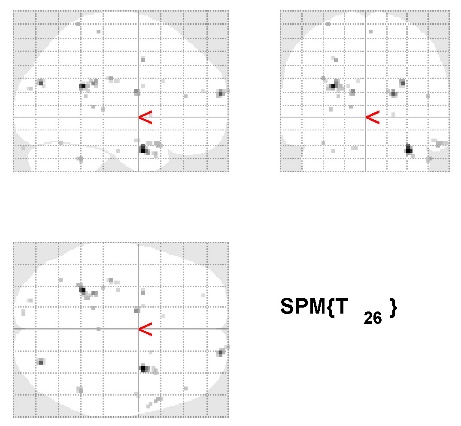 | 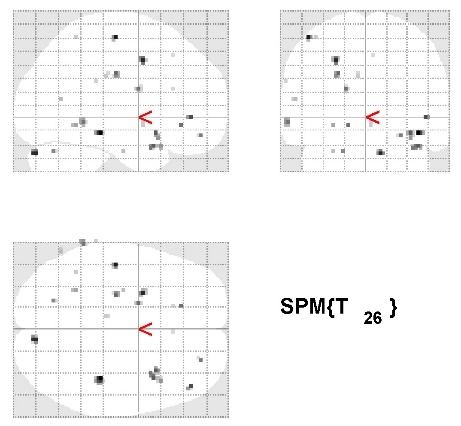 |
| Association PANAS negative and ALFF-functional asymmetry induction in rMDD | Association PANAS negative and fALFF-functional asymmetry induction in rMDD | Association PANAS negative and ReHo-functional asymmetry induction in rMDD |
| cluster-level: p_fwe_= 0.027, K_E_= 77.  peak-level: p_fwe_= 0.994, T= 5.04, MNI= -32 -28 6.  p_fwe_= 1.000, T= 3.60, MNI= -28 -22 12.  cluster-level: p_fwe_= 0.000, K_E_= 212.  peak-level: p_fwe_= 1.000, T= 4.74, MNI= -34 -20 28.  p_fwe_= 1.000, T= 4.48, MNI= -30 -14 34.  p_fwe_= 1.000, T= 4.35, MNI= -28 -24 42. |  |  |
| 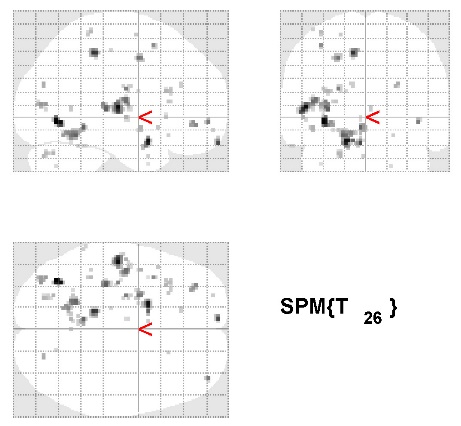 | 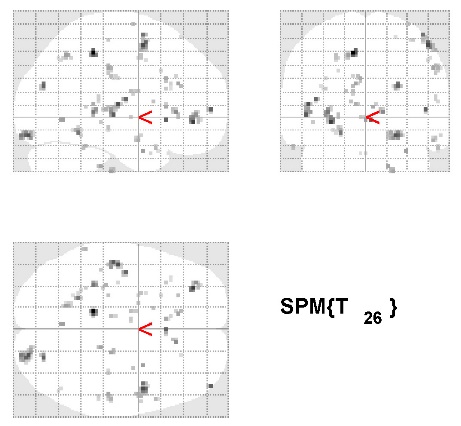 | 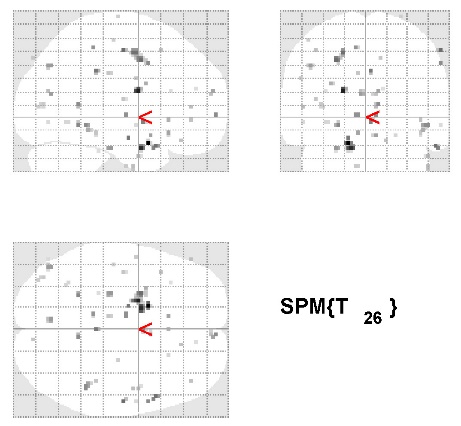 |
| Association PANAS negative and ALFF-functional asymmetry change in rMDD | Association PANAS negative and fALFF-functional asymmetry change in rMDD | Association PANAS negative and ReHo-functional asymmetry change in rMDD |
| cluster-level: p_fwe_= 0.026, K_E_= 48.  peak-level: p_fwe_= 1.000, T= 4.39, MNI= -20 -52 -16.  p_fwe_= 1.000, T= 4.10, MNI= -14 -58 -14. | cluster-level: p_fwe_= 0.025, K_E_= 42.  peak-level: p_fwe_= 1.000, T= 4.60, MNI= 22 -90 -16. |  |
| 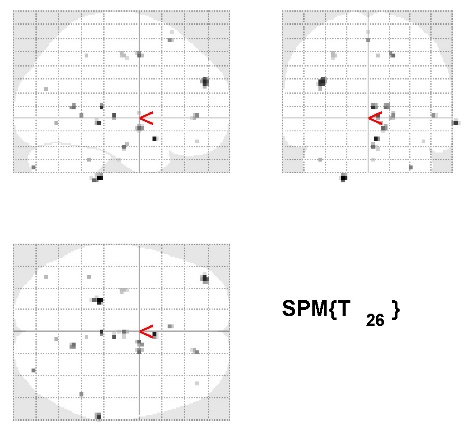 | 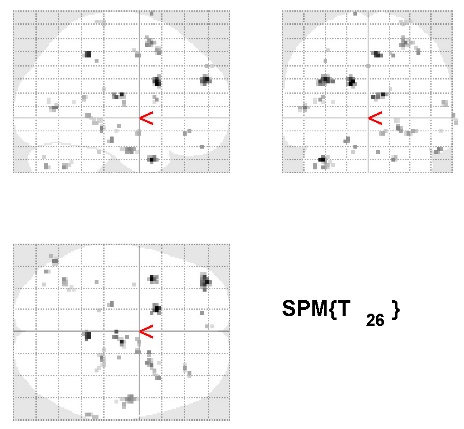 | 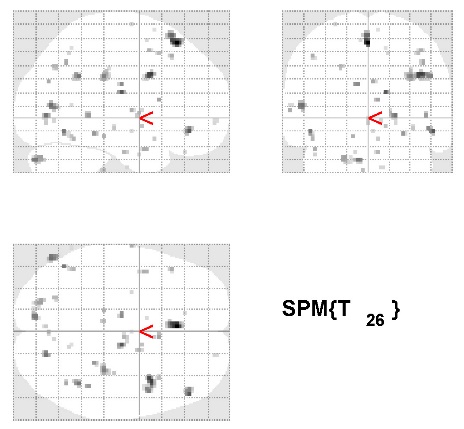 |
| Association PANAS positive and ALFF-functional asymmetry rest in rMDD | Association PANAS positive and fALFF-functional asymmetry rest in rMDD | Association PANAS positive and ReHo-functional asymmetry rest in rMDD |
|  |  |  |
| 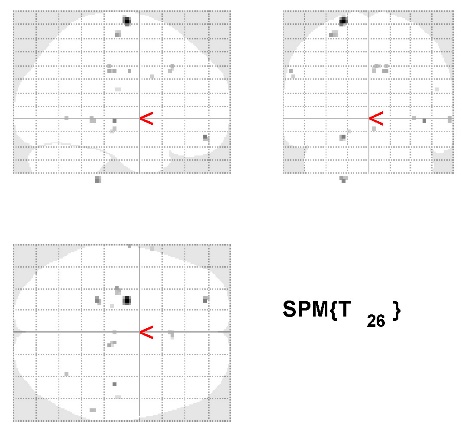 | 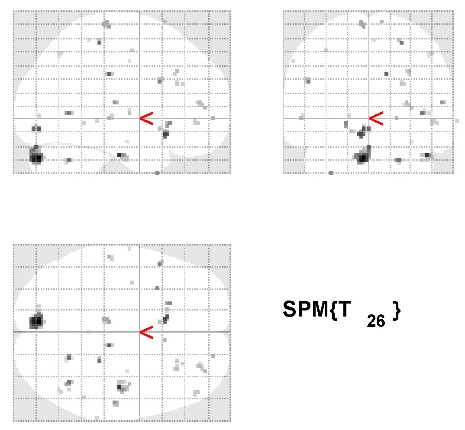 | 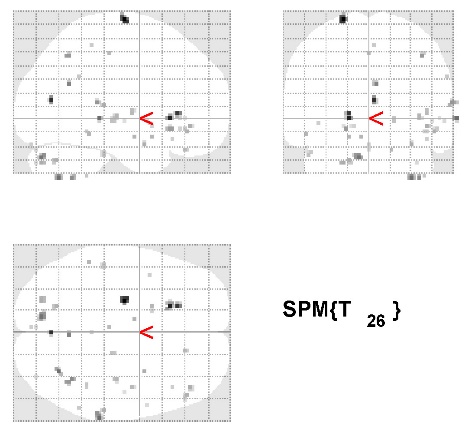 |
| Association PANAS positive and ALFF-functional asymmetry induction in rMDD | Association PANAS positive and fALFF-functional asymmetry induction in rMDD | Association PANAS positive and ReHo-functional asymmetry induction in rMDD |
|  | cluster-level: p_fwe_= 0.000, K_E_= 88.  peak-level: p_fwe_= 1.000, T= 5.03, MNI= -6 -82 -32.  p_fwe_= 1.000, T= 4.36, MNI= -2 -84 -24. |  |
| 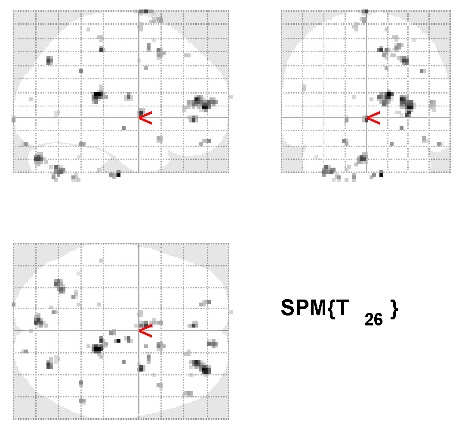 | 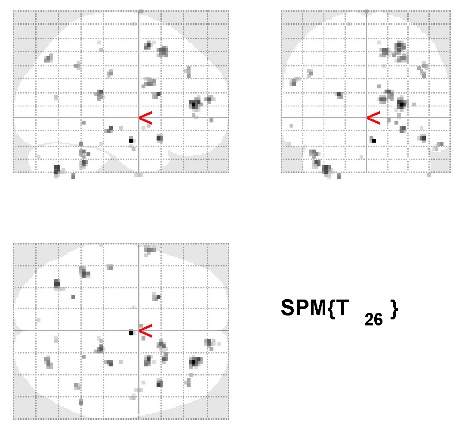 | 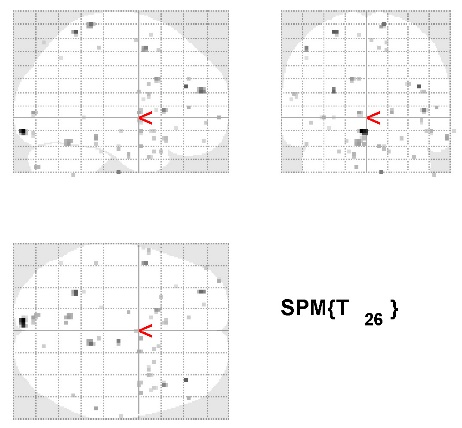 |
| Association PANAS positive and ALFF-functional asymmetry change in rMDD | Association PANAS positive and fALFF-functional asymmetry change in rMDD | Association PANAS positive and ReHo-functional asymmetry change in rMDD |
| cluster-level: p_fwe_= 0.003, K_E_= 68.  peak-level: p_fwe_= 1.000, T= 4.79, MNI= 32 50 6.  p_fwe_= 1.000, T= 4.66, MNI= 26 44 12. |  |  |

**
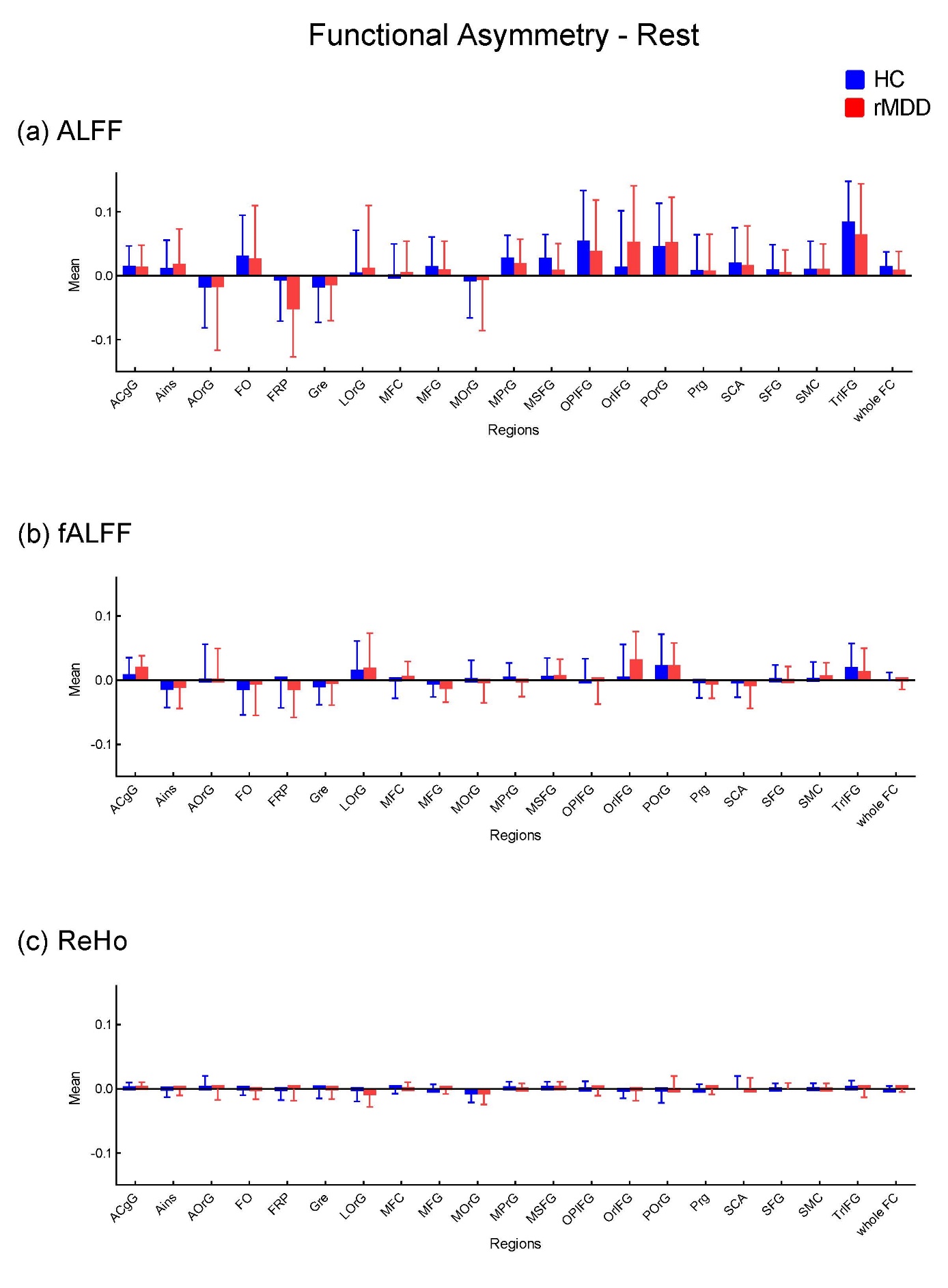
**
**Figure S1. Regional Functional Asymmetry in the Rest Phase.** Each bar presents the average value of the functional asymmetry of the region, the error bar is the standard error of the mean. Asymmetry index used: AI = (xl - xr)/(xl + xr) with subscripts l and r identifying the left and right instances of value x, AI becomes positive means higher activation in the left hemisphere, and AI becomes negative means higher activation in the right hemisphere. See the first part of the supplement for region abbreviations.


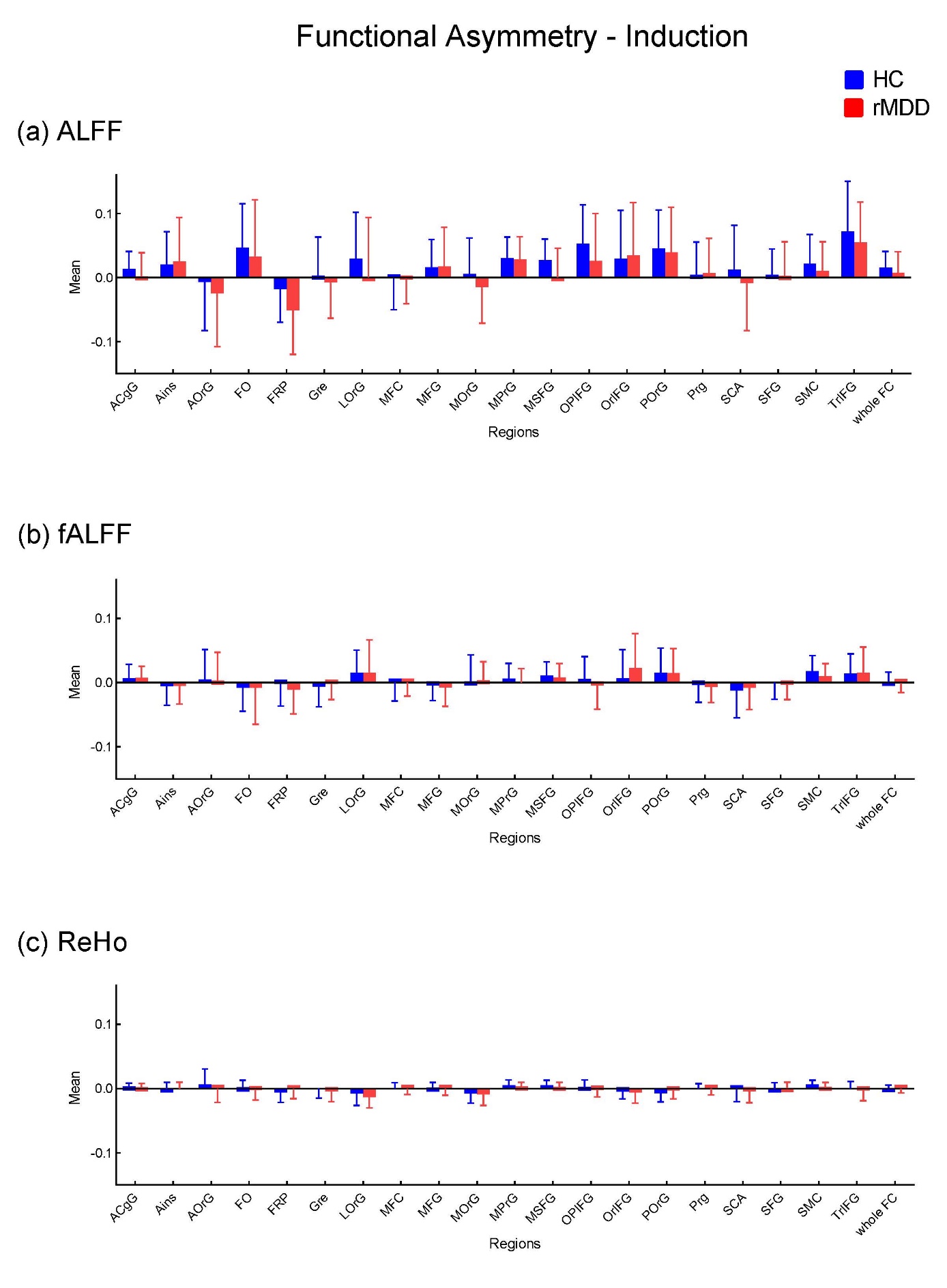


**Figure S2. Regional Functional Asymmetry in the Induction Phase.** Each bar presents the average value of the functional asymmetry of the region, the error bar is the standard error of the mean. Asymmetry index used: AI = (xl - xr)/(xl + xr) with subscripts l and r identifying the left and right instances of value x, AI becomes positive means higher activation in the left hemisphere, and AI becomes negative means higher activation in the right hemisphere. See the first part of the supplement for region abbreviations.


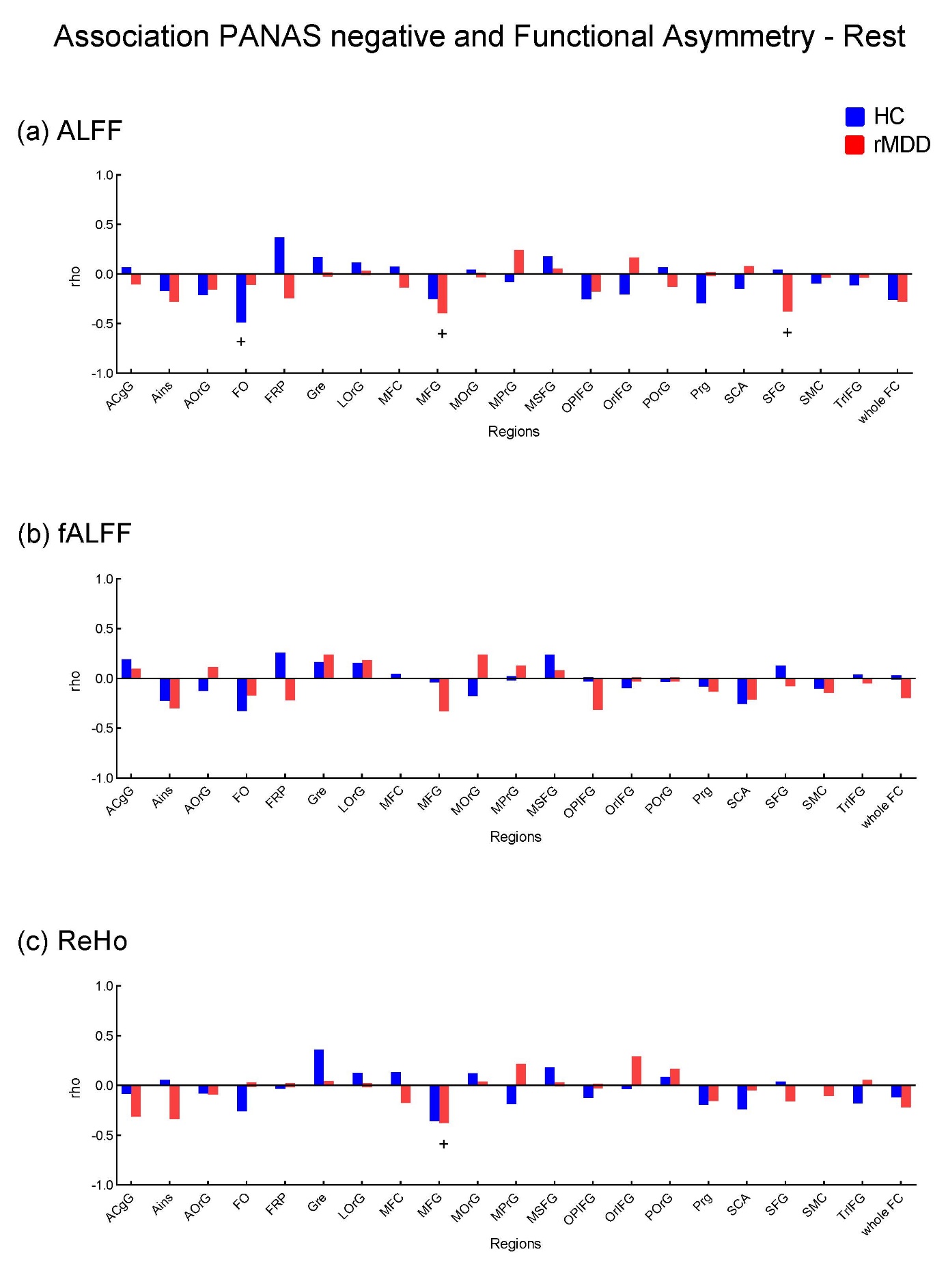


**Figure S3. Relationship between the PANAS Negative Score and Functional Asymmetry in the Rest Phase.** The bar chart presents the strength of the association(rho). See the first part of the supplement for region abbreviations. ＋p＜0.05.


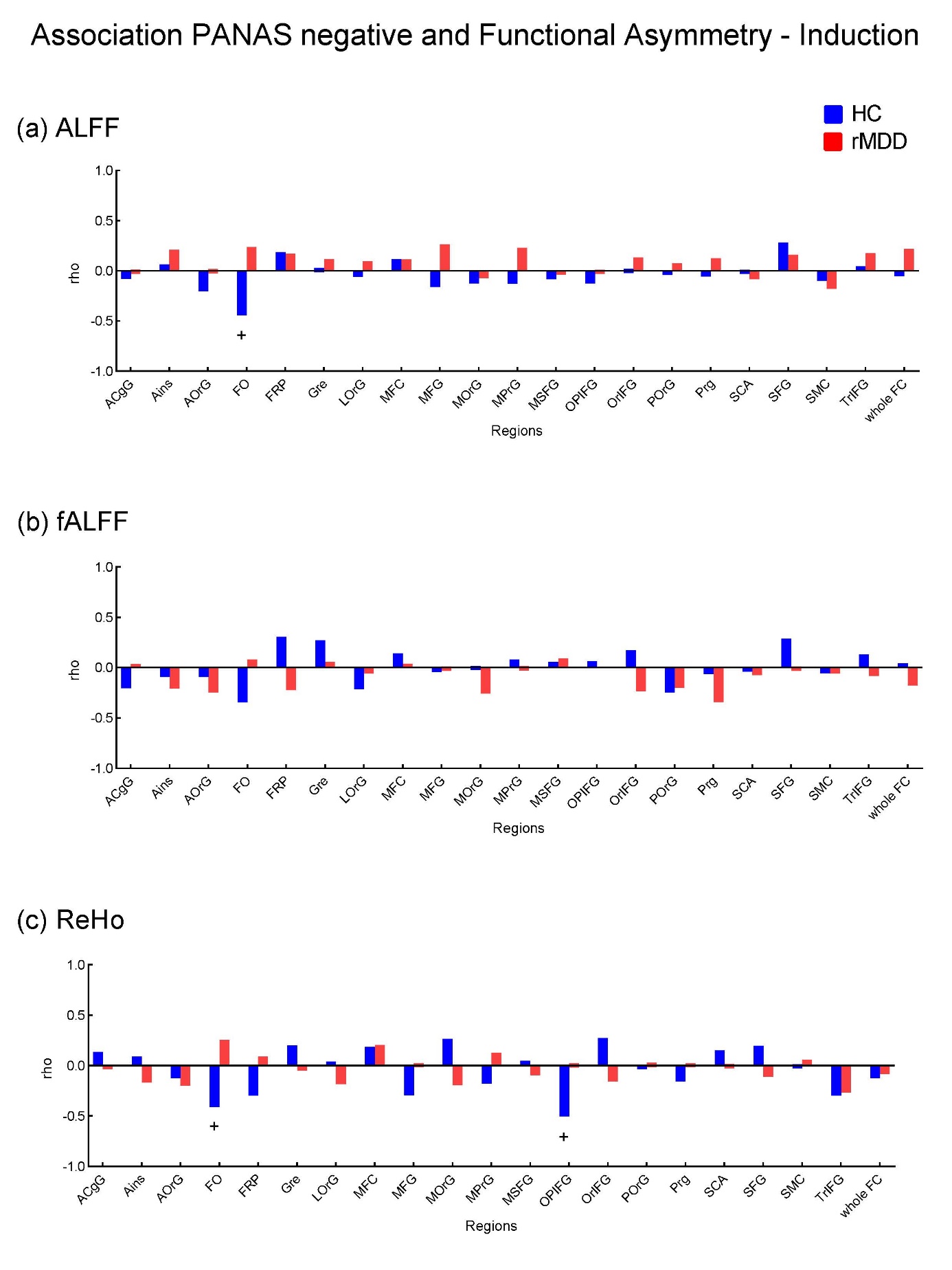


**Figure S4. Relationship between the PANAS Negative Score and Functional Asymmetry in the Induction Phase.** The bar chart presents the strength of the association(rho). See the first part of the supplement for region abbreviations. ＋p＜0.05.


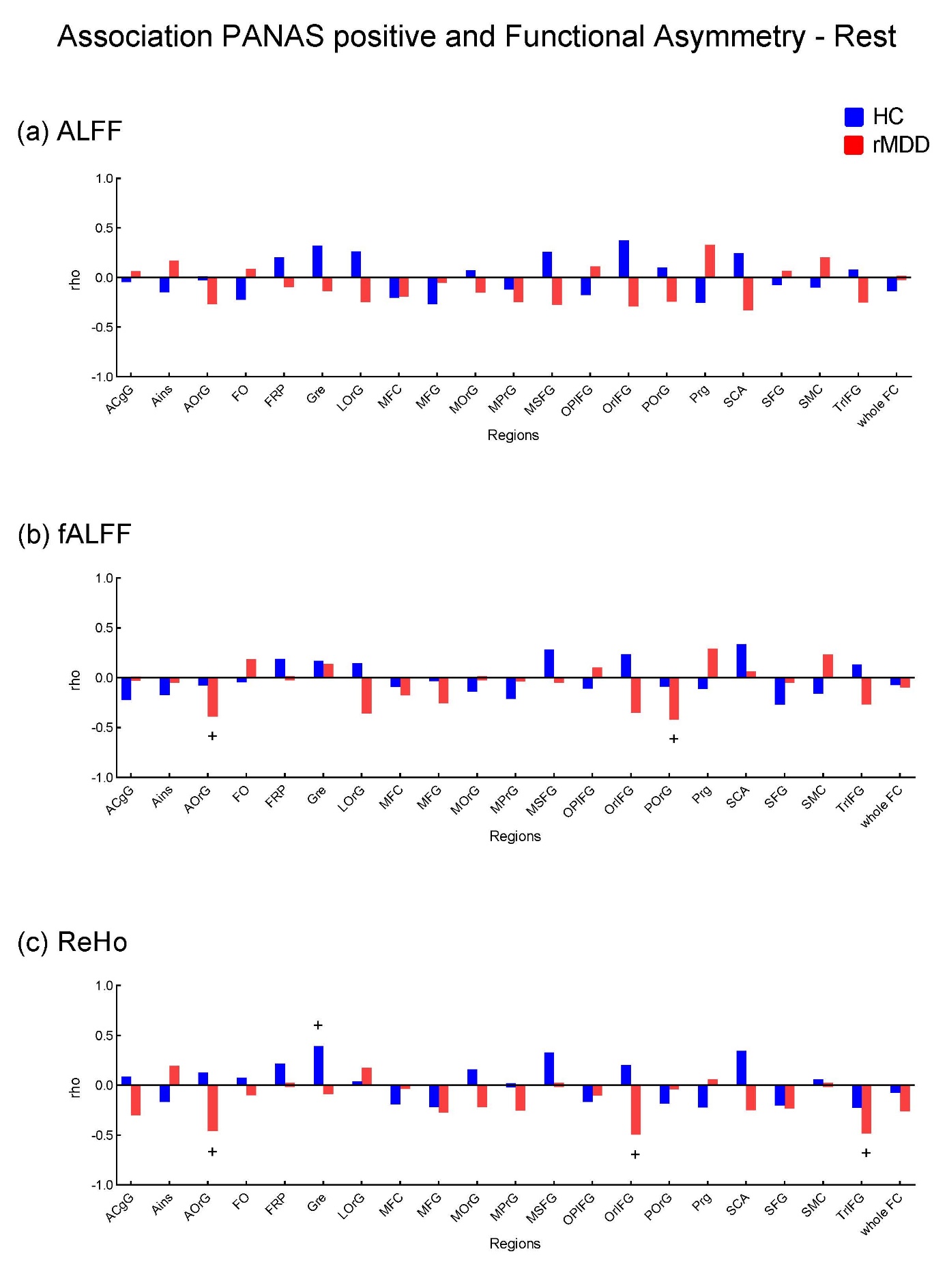


**Figure S5. Relationship between the PANAS Positive Score and Functional Asymmetry in the Rest Phase.** The bar chart presents the strength of the association(rho). See the first part of the supplement for region abbreviations. ＋p＜0.05.

**
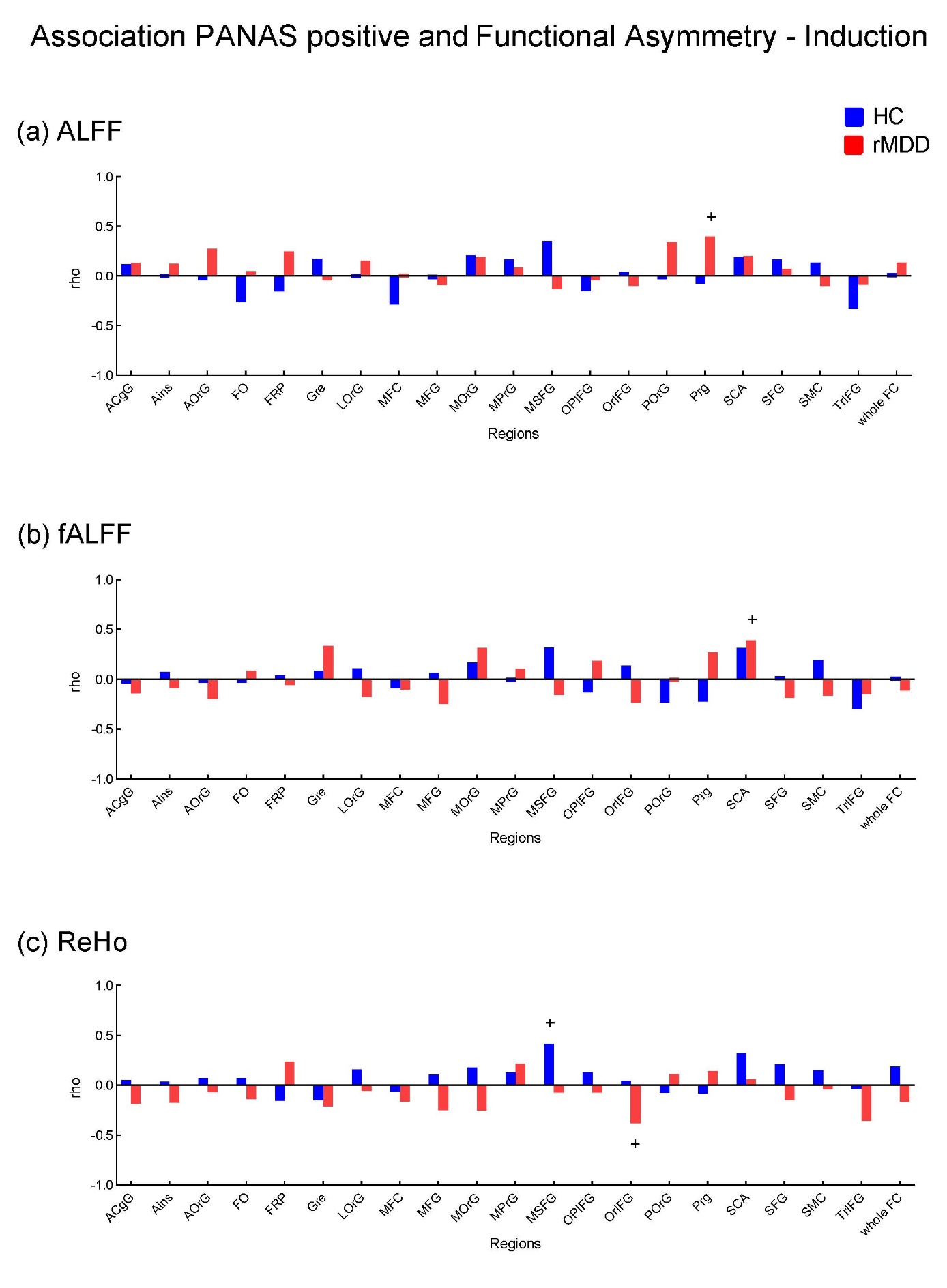
 Figure S6. Relationship between the PANAS Positive Score and Functional Asymmetry in the Induction Phase.** The bar chart presents the strength of the association(rho). See the first part of the supplement for region abbreviations. ＋p＜0.05.


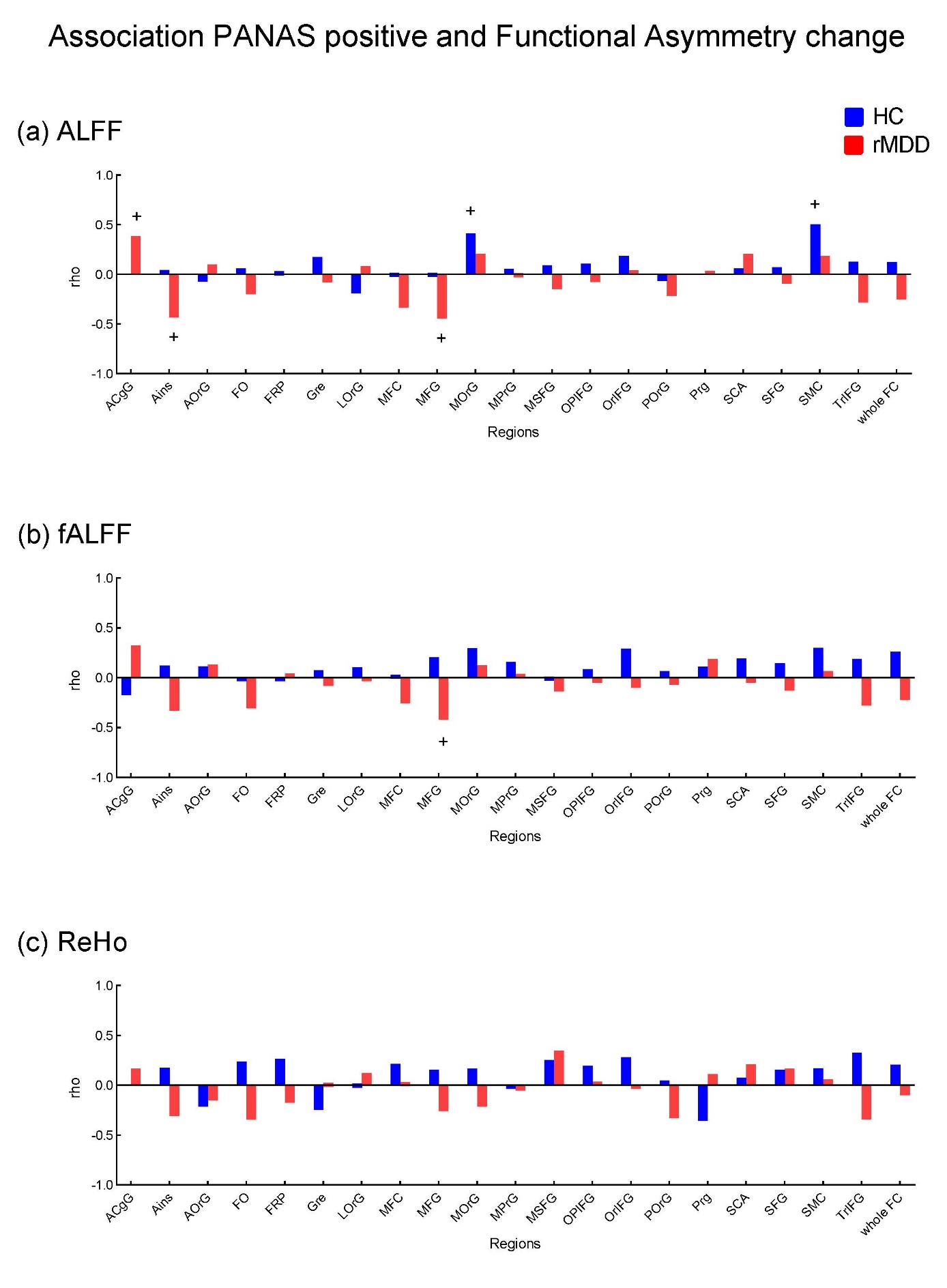


**Figure S7. Relationship between the Change in PANAS Positive Score and the Change in Functional Asymmetry.** The change is defined as Induction – Rest. The bar chart presents the strength of the association(rho). See the first part of the supplement for region abbreviations. ＋p＜0.05.

**
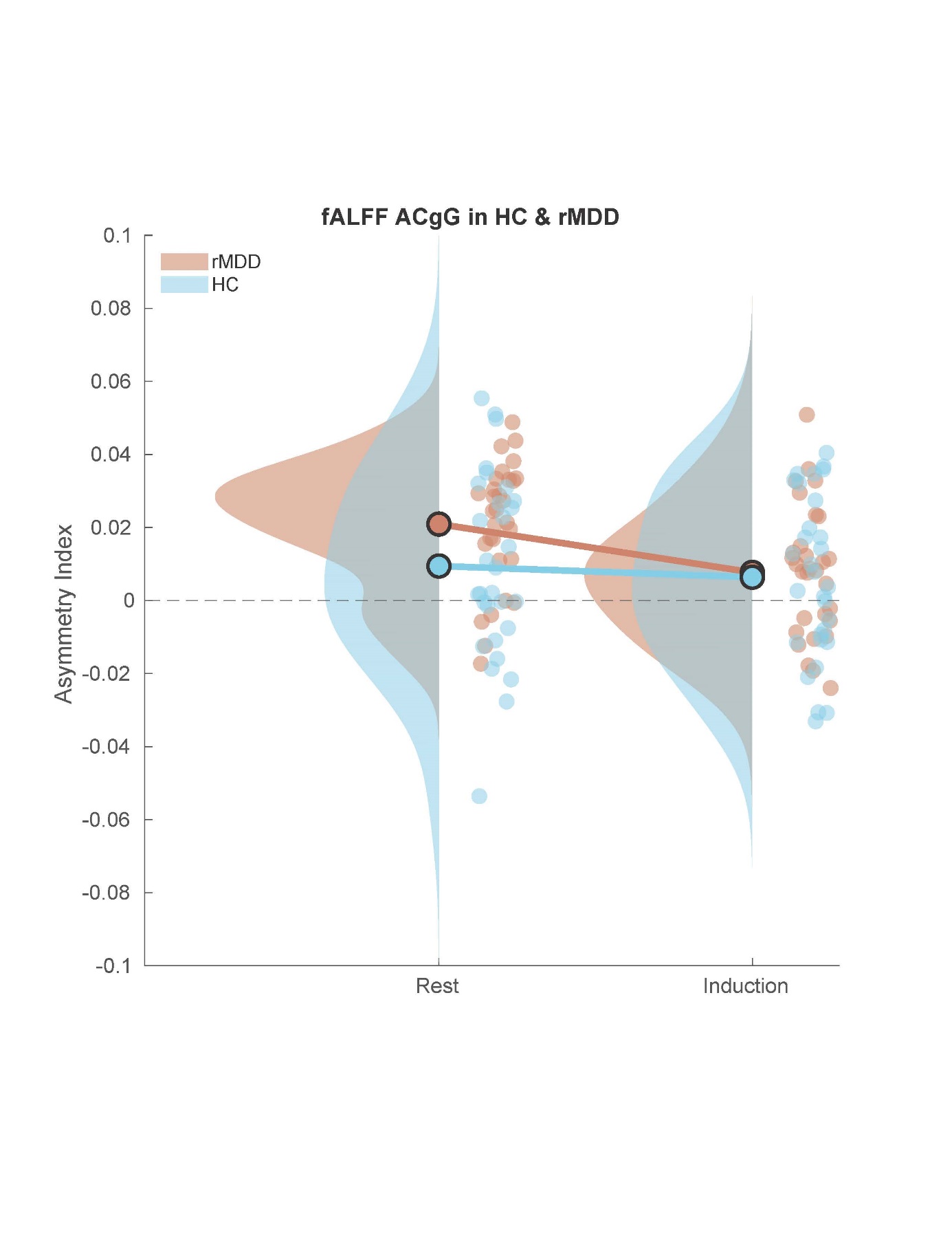
**

**Figure S8. fALFF Functional Asymmetry of the Anterior Cingulate Gyrus (ACgG) in the Rest Phase and Induction Phase.** The raincloud figure shows raw data (rain; each dot per Asymmetry Index (AI), AI used: (L − R)/(L + R)), probability density (cloud), and the mean of the raw data (the dot in the cloud). The solid line shows the change of the mean between the Rest phase and the Induction phase. This figure indicates that the decrease in rMDD (t(27)=-3.97, p=0.00048, g=-0.704), just let rMDD return to the same level of the HC.

**
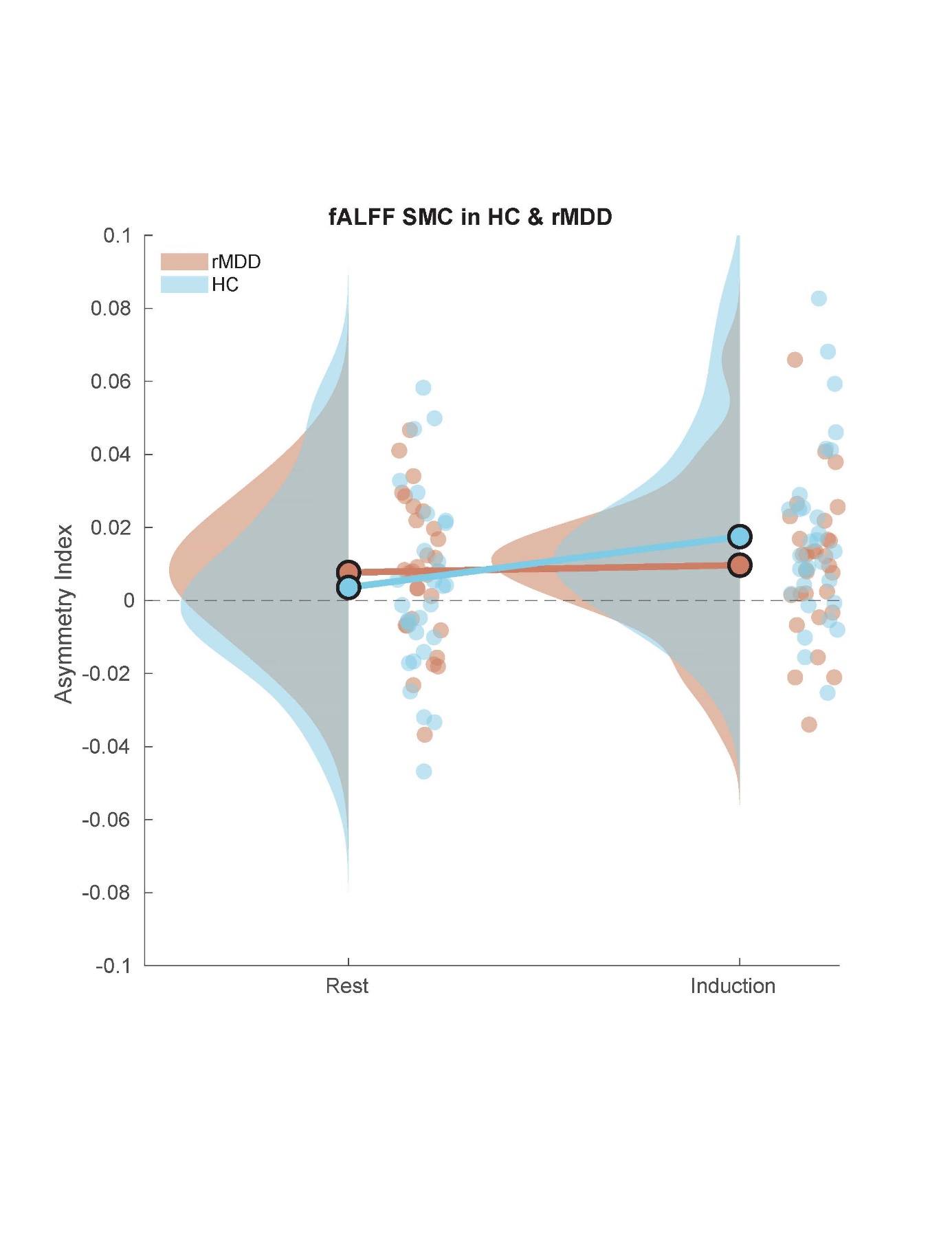
**

**Figure S9. fALFF Functional Asymmetry of the Supplementary Motor Cortex (SMC) in the Rest Phase and Induction Phase.** The raincloud figure shows raw data (rain; each dot per Asymmetry Index (AI), AI used: (L − R)/(L + R)), probability density (cloud), and the mean of the raw data (the dot in the cloud). The solid line shows the change of the mean between the Rest phase and the Induction phase. This figure indicates the fALFF functional asymmetry increased after the induction in the HC group, but not in the rMDD group.

**
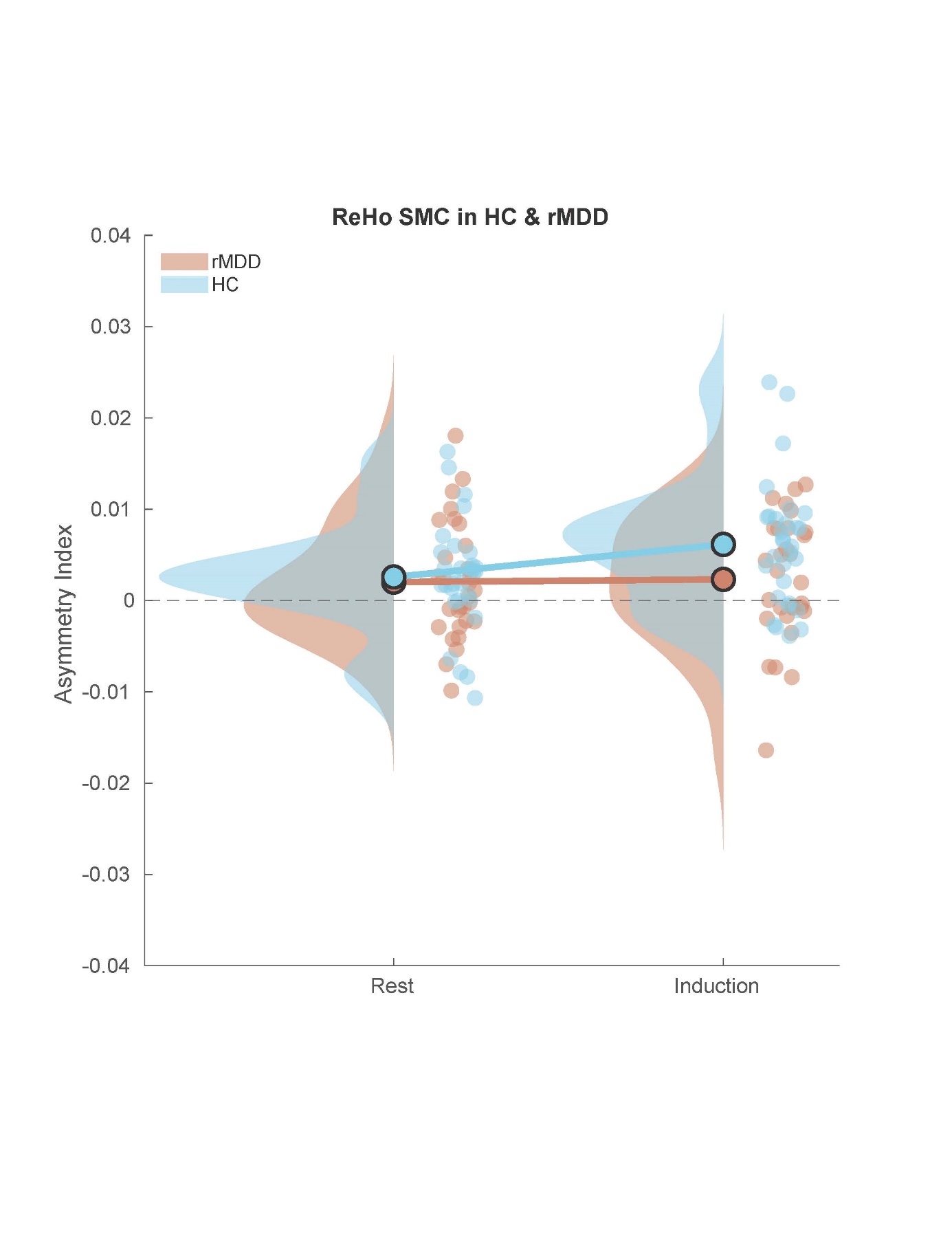
**

**Figure S10. ReHo Functional Asymmetry of the Supplementary Motor Cortex (SMC) in the Rest Phase and Induction Phase.** The raincloud figure shows raw data (rain; each dot per Asymmetry Index (AI), AI used: (L − R)/(L + R)), probability density (cloud), and the mean of the raw data (the dot in the cloud). The solid line shows the change of the mean between the Rest phase and the Induction phase. This figure indicates the lower ReHo functional asymmetry in SMC (t(56)=2.26, p=0.028, g=0.577) in rMDD, is based on an increase in the HC group while these values are not changed in the rMDD group.

**Table S3. Results of mixed ANOVA.**

|  |  | group effect | | time effect | | group * time | |
| --- | --- | --- | --- | --- | --- | --- | --- |
| variable/ROI | | F | p | F | p | F | p |
| PANAS - negative | | 5.025 | 0.029^＋^ | 31.040 | <0.001* | 9.846 | 0.003^＋^ |
| PANAS - positive | | 2.595 | 0.113 | 10.653 | 0.002* | 2.451 | 0.123 |
| ALFF: | |  |  |  |  |  |  |
| 1 | ACgG | 0.693 | 0.409 | 3.348 | 0.073 | 1.887 | 0.175 |
| 2 | AIns | 0.176 | 0.676 | 1.948 | 0.168 | 0.007 | 0.934 |
| 3 | AOrG | 0.174 | 0.678 | 0.169 | 0.683 | 0.332 | 0.567 |
| 4 | FO | 0.257 | 0.614 | 1.736 | 0.193 | 0.411 | 0.524 |
| 5 | FRP | 7.195 | 0.010^＋^ | 0.188 | 0.666 | 0.311 | 0.579 |
| 6 | GRe | 0.069 | 0.794 | 4.142 | 0.046^＋^ | 0.982 | 0.326 |
| 7 | LOrG | 0.320 | 0.574 | 0.378 | 0.541 | 3.221 | 0.078 |
| 8 | MFC | 0.012 | 0.915 | 0.930 | 0.339 | 0.235 | 0.630 |
| 9 | MFG | 0.026 | 0.872 | 0.521 | 0.473 | 0.294 | 0.590 |
| 10 | MOrG | 0.425 | 0.517 | 0.149 | 0.701 | 1.744 | 0.192 |
| 11 | MPrG | 0.373 | 0.544 | 1.586 | 0.213 | 0.790 | 0.378 |
| 12 | MSFG | 5.913 | 0.018^＋^ | 1.155 | 0.287 | 0.891 | 0.349 |
| 13 | OPIFG | 1.529 | 0.221 | 0.980 | 0.326 | 0.549 | 0.462 |
| 14 | OrIFG | 1.332 | 0.253 | 0.018 | 0.893 | 2.521 | 0.118 |
| 15 | POrG | 0.000 | 0.999 | 0.631 | 0.430 | 0.437 | 0.511 |
| 16 | Prg | 0.008 | 0.930 | 0.233 | 0.631 | 0.056 | 0.813 |
| 17 | SCA | 0.618 | 0.435 | 4.681 | 0.035^＋^ | 1.425 | 0.238 |
| 18 | SFG | 0.078 | 0.781 | 0.636 | 0.428 | 0.055 | 0.815 |
| 19 | SMC | 0.289 | 0.593 | 1.465 | 0.231 | 1.745 | 0.192 |
| 20 | TrIFG | 1.304 | 0.258 | 1.736 | 0.193 | 0.018 | 0.892 |
| 21 | whole frontal cortex | 1.045 | 0.311 | 0.062 | 0.805 | 0.117 | 0.734 |
| fALFF: | |  |  |  |  |  |  |
| 1 | ACgG | 2.201 | 0.143 | 8.224 | 0.006^＋^ | 3.365 | 0.072 |
| 2 | AIns | 0.057 | 0.813 | 3.953 | 0.052 | 0.115 | 0.736 |
| 3 | AOrG | 0.009 | 0.924 | 0.031 | 0.861 | 0.014 | 0.906 |
| 4 | FO | 0.189 | 0.665 | 0.206 | 0.651 | 0.527 | 0.421 |
| 5 | FRP | 2.125 | 0.150 | 0.054 | 0.817 | 0.197 | 0.659 |
| 6 | GRe | 0.595 | 0.444 | 0.897 | 0.348 | 0.021 | 0.885 |
| 7 | LOrG | 0.027 | 0.871 | 0.046 | 0.831 | 0.021 | 0.884 |
| 8 | MFC | 0.486 | 0.488 | 0.868 | 0.356 | 0.983 | 0.326 |
| 9 | MFG | 0.914 | 0.343 | 1.599 | 0.211 | 0.363 | 0.549 |
| 10 | MOrG | 0.178 | 0.674 | 0.539 | 0.466 | 0.754 | 0.389 |
| 11 | MPrG | 2.721 | 0.104 | 0.529 | 0.470 | 0.281 | 0.598 |
| 12 | MSFG | 0.051 | 0.822 | 0.389 | 0.535 | 0.319 | 0.575 |
| 13 | OPIFG | 1.194 | 0.279 | 0.160 | 0.691 | 0.115 | 0.735 |
| 14 | OrIFG | 3.830 | 0.055 | 0.590 | 0.446 | 0.837 | 0.364 |
| 15 | POrG | 0.010 | 0.919 | 1.863 | 0.178 | 0.008 | 0.927 |
| 16 | Prg | 0.181 | 0.672 | 0.002 | 0.962 | 0.001 | 0.981 |
| 17 | SCA | 0.002 | 0.967 | 0.423 | 0.518 | 0.506 | 0.480 |
| 18 | SFG | 0.229 | 0.634 | 1.709 | 0.196 | 0.075 | 0.785 |
| 19 | SMC | 0.153 | 0.697 | 6.091 | 0.017^＋^ | 3.414 | 0.070 |
| 20 | TrIFG | 0.216 | 0.644 | 0.152 | 0.698 | 0.334 | 0.565 |
| 21 | whole frontal cortex | 0.333 | 0.566 | 0.072 | 0.789 | 0.010 | 0.920 |
| ReHo: | |  |  |  |  |  |  |
| 1 | ACgG | 0.003 | 0.957 | 3.776 | 0.057 | 1.645 | 0.205 |
| 2 | AIns | 0.092 | 0.763 | 2.910 | 0.093 | 0.268 | 0.607 |
| 3 | AOrG | 2.327 | 0.133 | 0.249 | 0.619 | 0.094 | 0.760 |
| 4 | FO | 1.448 | 0.234 | 1.265 | 0.266 | 0.482 | 0.490 |
| 5 | FRP | 1.399 | 0.242 | 0.644 | 0.426 | 0.272 | 0.604 |
| 6 | GRe | 0.633 | 0.429 | 0.075 | 0.786 | 0.467 | 0.497 |
| 7 | LOrG | 1.596 | 0.212 | 2.061 | 0.157 | 0.030 | 0.864 |
| 8 | MFC | 0.532 | 0.469 | 1.089 | 0.301 | 1.702 | 0.197 |
| 9 | MFG | 1.298 | 0.260 | 0.978 | 0.327 | 0.017 | 0.898 |
| 10 | MOrG | 0.096 | 0.758 | 0.003 | 0.959 | 0.121 | 0.729 |
| 11 | MPrG | 1.605 | 0.210 | 1.219 | 0.274 | 0.001 | 0.977 |
| 12 | MSFG | 0.804 | 0.374 | 0.140 | 0.709 | 1.267 | 0.265 |
| 13 | OPIFG | 3.178 | 0.080 | 0.023 | 0.880 | 0.125 | 0.725 |
| 14 | OrIFG | 0.035 | 0.853 | 0.513 | 0.477 | 0.413 | 0.523 |
| 15 | POrG | 1.990 | 0.164 | 2.496 | 0.120 | 0.049 | 0.825 |
| 16 | Prg | 0.134 | 0.716 | 0.021 | 0.885 | 0.017 | 0.896 |
| 17 | SCA | 0.075 | 0.785 | 1.430 | 0.237 | 0.338 | 0.563 |
| 18 | SFG | 0.244 | 0.623 | 0.140 | 0.710 | 0.782 | 0.380 |
| 19 | SMC | 2.207 | 0.143 | 4.707 | 0.034^＋^ | 3.381 | 0.071 |
| 20 | TrIFG | 2.907 | 0.094 | 2.623 | 0.111 | 0.120 | 0.730 |
| 21 | whole frontal cortex | 1.300 | 0.259 | 0.005 | 0.946 | 0.048 | 0.827 |

^＋^p<0.05, *p<0.0024;

See the first part of the supplement for region abbreviations.
